# Functional memory of drought affects leaf chemical defenses and microbial interactions in aspen

**DOI:** 10.1111/nph.71561

**Published:** 2026-09-13

**Authors:** Aubrey M. Hawks, Jaycie C. Fickle, Kelly L. Kerr, Dale L. Forrister, Allison M. Perkins, Efthymia Symeonidi, Madelyn Allen, Cannon Fehrenbach, Hsiao‐Nung Chen, Richard L. Lindroth, William R. L. Anderegg, Talia L. Karasov

**Affiliations:** ^1^ School of Biological Sciences University of Utah Salt Lake City UT 84112 USA; ^2^ Oak Ridge Institute for Science and Education, Rocky Mountain Research Station, USDA Forest Service Fort Collins CO 80526 USA; ^3^ Biodiversity Center University of Texas at Austin Austin TX 78712 USA; ^4^ Department of Chemistry University of Utah Salt Lake City UT 84112 USA; ^5^ Department of Entomology University of Wisconsin Madison WI 53706 USA

**Keywords:** aspen, chemical defense, drought, fungi, legacy, microbiome

## Abstract

- Drought is an important driver of tree mortality, but its long‐term impacts on trait expression remain poorly understood. Leaves are a key interface at which trees respond to abiotic stress, and foliar traits such as chemical defenses and microbial communities may play a role in determining post‐drought resilience. We tested whether prior‐year drought leaves a persistent imprint on leaf traits in *Populus tremuloides* Michx*.*
- Using a 3‐year common garden experiment with controlled water limitation, we measured two classes of defensive phenolics—salicinoid phenolic glycosides (SPGs) and condensed tannins (CTs)—alongside foliar fungal community composition.
- SPG concentrations increased and CTs declined in response to prior‐year drought, with no evidence of a trade‐off between increased allocation to defensive chemistry and growth. Higher SPG concentrations were associated with reduced canopy damage across treatments, while CT‐damaged canopy relationships depended on prior‐year drought conditions. The relative abundance of pathogen‐capable fungi increased under drought but declined with increasing SPG concentrations, consistent with the possibility that drought legacy effects on foliar chemistry extend to microbial colonization.
- These results indicate that drought‐induced shifts in foliar chemistry can persist beyond the drought period and may continue to influence plant performance and biotic interactions, with implications for post‐drought resilience.

## Graphical abstract

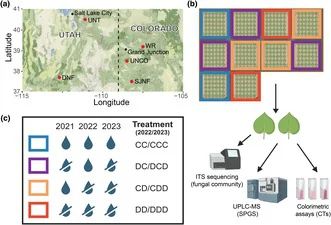

## Introduction

Hotter, drier, and more prolonged droughts linked to climate change are now recognized as a dominant driver of tree mortality, with impacts that can extend for years after drought conditions subside (Brodribb *et al*., 2020; Hammond *et al*., 2022; Gazol *et al*., 2025). Despite advances in understanding many of the physiological factors contributing to tree mortality, significant gaps remain between model predictions and observed patterns of forest die‐off (Trugman *et al*., 2021; Hartmann *et al*., 2022). Two factors are increasingly recognized as central to this limitation: high variation at the level of individual trees, such as genetic variation in stress‐related traits, differences in past stress exposure or microclimate, and interactions with biotic agents, including foliar pests and pathogens (Anderegg *et al*., 2015; Hartmann *et al*., 2018; Trugman *et al*., 2021). Because foliar traits mediate interactions with both abiotic stressors and biotic agents, they may be critical for understanding drought‐driven mortality. Among these, defensive chemistry and foliar microbial communities stand out as traits that directly influence how trees interact with pests, pathogens, and stress (Nakabayashi & Saito, 2015; Trivedi *et al*., 2020). These dynamics are insufficiently represented in current models, largely because we lack mechanistic understanding of how drought alters tree–biotic interactions and how those interactions amplify or dampen mortality risk (Huang *et al*., 2020). Key unknowns are how drought reshapes leaf chemistry and microbial communities, how persistent these changes are, and whether these factors contribute to tree resilience or vulnerability post‐drought.

Leaves are a critical interface where trees encounter both abiotic stress and biotic agents, and traits expressed there are likely to mediate how drought influences tree performance. Plants invest substantial resources in specialized metabolites – up to 5% of their photosynthate and as much as 20% of available nitrogen (Gleadow *et al*., 1998; Burns *et al*., 2002; Huang *et al*., 2019) – despite the associated costs to growth, because these compounds improve stress tolerance and defense (Cole *et al*., 2016; Cope *et al*., 2021). By influencing herbivores, microbial colonization, litter decomposition, and nutrient cycling, these compounds generate cascading ecological effects (Schweitzer *et al*., 2008; Rubert‐Nason & Lindroth, 2021; Kelly *et al.*, 2010; Winder *et al.*, 2013). Many of the most abundant plant metabolites are antioxidants that mitigate abiotic stress directly by scavenging reactive oxygen species during drought (Munné‐Bosch & Alegre, 2000; Nakabayashi & Saito, 2015; Gourlay *et al*., 2022; Moroldo *et al*., 2024). Foliar microbes, in turn, can shape tree health by enhancing stress tolerance or, conversely, by acting as opportunistic pathogens (Trivedi *et al*., 2020; Cambon *et al*., 2022; Singh *et al*., 2023). Their composition reflects both environmental filtering and host traits, including chemical defenses, positioning chemistry and microbes as potentially intersecting mediators of drought outcomes and community composition (Forrister *et al*., 2019).

Evidence that drought influences allocation to specialized metabolites comes from a wide range of studies, with stronger experimental support in crop species than in trees. Shifts in terpenes and phenolics have been measured following short periods of drought, though the magnitude and direction of responses vary with drought severity and plant species (Koricheva *et al*., 1998; Munné‐Bosch & Alegre, 2000; Gerson & Kelsey, 2004; Nogués *et al*., 2014; Rivas‐Ubach *et al*., 2014; Kleiber *et al*., 2017; Piasecka *et al*., 2017; Shamloo *et al*., 2017; Holopainen *et al*., 2018; Sarker & Oba, 2018; Khaleghi *et al*., 2019). In conifers, such shifts can have direct ecological consequences; for example, decreases in monoterpene concentrations in *Pinus edulis* have been associated with increased bark beetle attack during drought (Gaylord *et al*., 2013; Thompson *et al*., 2024; Malone *et al*., 2025). More recent research suggests the potential for longer term impacts of severe drought stress. Laoué *et al*. (2024) found reduced flavanol allocation in *Quercus pubescens* that persisted for up to 2 yr, and (Eisenring *et al*., 2024) documented lasting metabolomic shifts in *Fagus sylvatica* following drought‐induced defoliation.

In deciduous trees, drought carry‐over effects are particularly intriguing because the foliage directly exposed to drought is shed and replaced each year. Thus, persistence in this context does not imply an indefinite response, but rather that drought‐associated changes remain detectable in newly produced leaves during subsequent growing seasons after the original drought‐affected foliage has been replaced. The above‐mentioned studies, conducted under field and semi‐natural conditions, indicate that drought may have a persistent effect on defensive chemistry. Replicated and randomized experiments are needed to assess whether drought consistently reshapes defensive chemistry, to clarify the mechanisms driving these responses, and to determine whether such changes translate into altered biotic interactions and ultimately, performance outcomes.

Evidence that drought alters plant‐associated microbial communities is accumulating rapidly, and water availability is emerging as a major determinant of the leaf microbiome (Chen *et al*., 2021; Zhu *et al*., 2022; Karasov *et al*., 2024). These effects are less well documented in forest trees, though rainfall‐exclusion experiments in *Quercus ilex* have reported increased bacterial and fungal diversity in the phyllosphere following drought (Peñuelas *et al*., 2012; Rico *et al*., 2014). In *Populus deltoides*, drought altered microbial communities in a sex‐dependent manner, with female trees hosting assemblages more resilient to pathogen infection (Lin *et al*., 2023). Shifts have also been reported in conifers, where changes in bacterial communities were observed under rain exclusion (Khlifa *et al*., 2025). Longer term drought effects on forest microbial communities have been documented in soils, but their persistence in foliage is much less certain (Hopkins *et al*., 2024; Jaeger *et al*., 2024). Moreover, the links between drought, foliar chemistry, and microbial colonization remain poorly understood.

We examined how drought history shapes specialized metabolism and foliar microbial communities using quaking aspen (*Populus tremuloides* Michx.), a widely distributed and chemically well‐characterized model for studying plant defense and plant–microbe interactions in forest ecosystems. Aspen produce two major classes of phenolic specialized metabolites – salicinoid phenolic glycosides (SPGs) and condensed tannins (CTs) – which are involved in both biotic and abiotic interactions. SPGs are salicylate derivatives largely restricted to the Salicaceae (e.g. *Populus* and *Salix*), while CTs (also known as proanthocyanidins) are flavan‐3‐ol polymers found broadly across plant taxa (Rubert‐Nason & Lindroth, 2021). In aspen, allocation to SPGs and CTs varies widely. Each can range from < 1% to over 30% of leaf dry weight. Both are products of the phenylpropanoid pathway, and their concentrations are often inversely correlated, suggesting a resource‐based trade‐off driven by shared biosynthetic precursors.

SPGs are strongly associated with herbivore deterrence: for example, each 1% increase in SPG leaf dry weight reduces female spongy moth larval weight by *c*. 73.9 mg (0.5%) and extends development time by 1.1 d (Hemming & Lindroth, 1995). Though they have been shown to reduce food preference in chrysomelid beetles, CTs are thought to have limited effects on herbivores. Their associations with herbivores are occasionally positive and may be explained by higher levels of SPGs in individuals with lower allocation to CTs (Gruppe *et al*., 1999; Rubert‐Nason & Lindroth, 2021).

Less is known about how these compounds influence microbial communities in Salicaceae. Existing work has focused primarily on specific pathogen systems, particularly rust fungi in the genus *Melampspora*. Elevated SPG concentrations have been associated with reduced infection severity in *Salix myrsinifolia* although evidence remains mixed (Hakulinen, 1998; Hakulinen & Julkunen‐Tiitto 2008; Maupetit *et al*., 2018). Across plant species, CTs have been found to be antimicrobial (Li *et al*., 2022; Ewing *et al*., 2024; Gupta *et al*., 2025; Mattos *et al*., 2025), though direct evidence in Salicaceae is more limited. Experimental work has shown that they can inhibit *Melampspora larici‐populina*; at 0.5 mg ml^−1^ of CTs reduced spore germination by 4.2‐fold *in vitro* (Ullah *et al*., 2017). More recently, bacterial communities in *Populus nigra* tissues with higher concentrations of secondary metabolites were found to be enriched for traits that may be related to tolerance to the chemical environment (Bose *et al*., 2025).

Natural surveys and experimental manipulations of foliar communities in *Populus* suggest that microbial assemblages are shaped by environmental conditions, with drier sites often harboring distinct community profiles (Lin *et al*., 2023; Van Nuland *et al*., 2023). These shifts may arise through multiple, nonmutually exclusive mechanisms, including direct effects of abiotic stress on microbial survival and colonization, as well as indirect, host‐mediated changes in leaf traits. If microbial taxa differ in their responses to phenolic compounds, host chemistry could act as a filter on microbial communities, such that shifts in chemical investment under environmental stress could influence community restructuring. Moreover, if drought‐induced chemical changes persist across years, they may continue to shape microbial communities after drought has ended.

We utilized a 3‐yr common garden drought experiment established by Kerr *et al*. to test whether drought leaves a legacy in phenolic chemistry, whether these changes have functional consequences for aspen performance and whether drought exerts direct or indirect effects on foliar microbial communities (Fig. 1; Kerr *et al*., 2023; Fickle *et al*., 2025). From 2021 to 2023, two blocks each were maintained under consistent control (CCC) or drought (DDD) conditions. An additional three blocks each simulated shorter drought periods (CDD) or recovery years between droughts (DCD; Fig. 1C). We sampled leaves at peak drought each year to address the following questions: (1) Does drought alter the chemical profile of aspen leaves, and do these effects persist into subsequent growing seasons? (2) Are drought‐associated changes in foliar chemistry associated with variation in growth and canopy condition? (3) Do contrasting drought histories affect foliar microbial communities and (4) Are drought‐associated shifts in SPG or CT concentrations linked to differences in foliar fungal community composition?

**Fig. 1:**
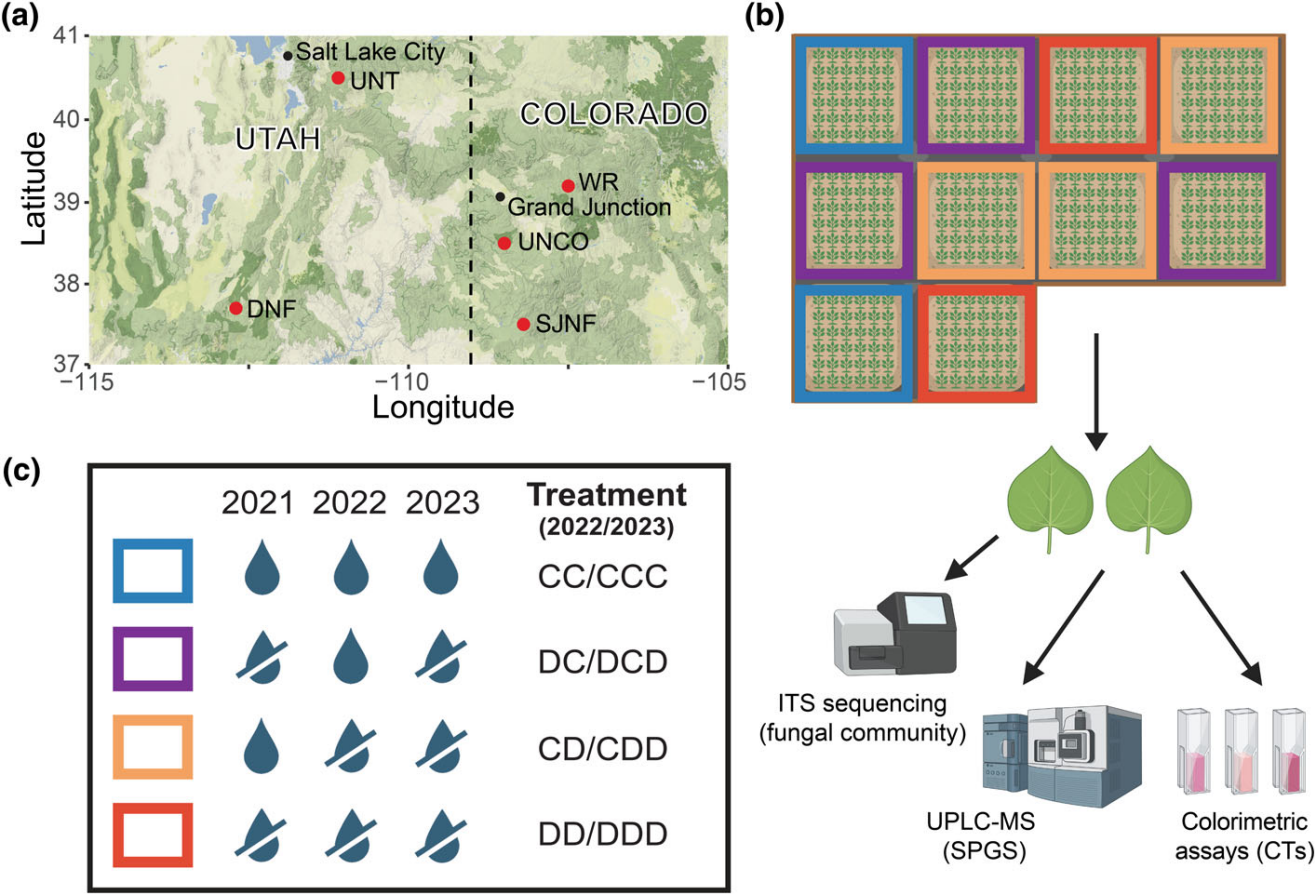
Experimental design of the common garden study. Field sites were established at locations in the Dixie (DNF), San Juan (SJNF), Uncompahgre (UNCO), White River (WR), and Uinta (UNT) National Forests across Utah and Colorado, USA (a). Red points mark site locations. Root stock collected from multiple stands of *Populus tremuloides* at each site was propagated and planted into randomized common garden blocks (b). From 2021 to 2023, trees in the common garden received either control or drought treatments from June to September (c). Paired samples were collected for analysis of specialized metabolites and microbial communities (b). Treatment histories are indicated by letter codes, where each letter represents a single year (C, control; D, drought), as shown by water icons. Labels are presented as paired abbreviations (e.g. DC/DCD), where the first term (DC) denotes the 2‐yr treatment history before the 2022 sampling (2021–2022) and the second term (DCD) indicates the full 3‐yr treatment history for the 2023 sampling (2021–2023). Colors and abbreviations are used consistently across subsequent figures. Parts of (b) and (c) were created with BioRender (Karasov, T. (2026) https://BioRender.com/h23yjqv).

## Materials and Methods

### Experimental design

Our study was conducted within the clonal quaking aspen (*Populus tremuloides* Michx.) field–common garden experiment established by Kerr *et al*. (2023) and further characterized by Fickle *et al*. (2026). This study system combines long‐term observations of natural populations with controlled drought manipulations applied in a common garden to propagated clones derived from the same individuals (Fig. 1). We used this framework to examine how contrasting drought histories shape foliar chemistry and fungal communities. Additional details regarding genotype origin, propagation, garden establishment, and drought treatment implementation are provided in Supporting Information Methods S1 and in Kerr *et al*. (2023).

### Field sites

Field sites were identified in the Dixie (DNF), San Juan (SJNF), Uncompahgre (UNCO), White River (WR), and Uinta (UNT) National Forests using the United States Forest Service Forest Inventory and Analysis (FIA) Database. Site selection followed Kerr *et al*. (2023), targeting a natural gradient in mean annual temperature (2.4–5.0°C) and precipitation (338–662 mm) while minimizing variation in elevation (*c*. 3000 m) and aspect (south‐facing).

At each site, plots were established on uniform slopes of similar steepness to reduce topographic effects on soil moisture. A central GPS reference point was established within each clonal stand, and five mature trees were selected for long‐term monitoring within an 18 m radius. Genotypic identity was verified using PCR amplification of eight microsatellite markers (Mock *et al*., 2012).

### Common garden

Clonal genotypes derived from field‐collected rootstock were propagated and established in a common garden at the University of Utah in 2020 (Kerr *et al*., 2023). The garden consisted of 10 randomized blocks, each containing a 6 × 6 grid of plants spaced 50 cm apart (360 individuals total). Genotypes were spatially randomized across blocks.

In 2021, blocks were randomly assigned to control or drought treatments. After an initial establishment period under full watering, drought treatments received reduced irrigation (50% then 25% of control) to achieve target predawn water potentials of *c*. −1.5 MPa, corresponding to moderate to severe drought stress (Love *et al*., 2019). Treatment assignments were modified in subsequent years to generate variation in drought history. In 2022, a subset of blocks retained their initial treatment, while others were switched to simulate recovery or short‐term drought (Fig. 1). In 2023, all noncontrol blocks received drought treatment. Because the experiment was designed to emphasize responses to changing water availability, treatment trajectories involving transitions between DCD and CDD were represented by three blocks each, whereas continuously watered (CCC) and repeated drought (DDD) trajectories were represented by two blocks each.

Because natural summer rainfall reduced drought severity in 2021, rain exclusion was implemented in 2022–2023 using temporary overhead canopies to maintain treatment separation. Canopies were placed over drought beds only during rainfall events and removed once precipitation ceased, thereby limiting potential microclimatic effects associated with permanent rain shelters. Drought intensity was monitored using weekly measurements of predawn and midday leaf water potential (Fickle *et al*., 2026).

### Chemical quantification

#### Leaf sampling and processing

Leaves for quantification of phenolic compounds were collected from the common garden in late August 2022 and 2023 at peak drought stress before senescence. All sampling was completed within 90 min at ambient temperatures below 30°C, and leaves were shaded during collection. One to two mature leaves per plant were sampled from mid‐canopy height. Leaves were selected to be representative of each individual and were not chosen based on visible disease symptoms. Samples were dried on silica gel in the dark at 25°C for 48 h, followed by vacuum centrifugation at 30°C for ≥ 12 h. Dried samples were stored on silica gel in airtight bags at −20°C until analysis. Leaves were transferred to 2‐ml tubes and homogenized with glass beads using a FastPrep system (4 m s^−1^, 20 s). Subsamples (*c*. 15 mg) were weighed for SPG and CT quantification.

#### SPG extraction and quantification

SPGs were extracted in methanol containing 0.01% β‐resorcylic acid and quantified via UPLC‐MS following Rubert‐Nason *et al*. (2017). Analytical standards for salicortin and tremulacin were provided by R. Lindroth and purified as described in Rubert‐Nason *et al*. (2018). Salicin was obtained commercially (≥ 99% purity; cat. no.: S0625; Sigma‐Aldrich).

Peak detection and integration were performed in MZmine v.4.3 via mzWizard using the following parameters: noise threshold (MS1) = 1E3; minimum feature height = 3.0E3; *m*/*z* tolerance = 0.02 (scan‐to‐scan), 0.015 (intra‐sample), and 0.02 (sample‐to‐sample); retention time range = 0.03–22.50 min; maximum peaks per chromatogram = 15; minimum consecutive scans = 4; approximate feature FWHM = 0.05 min; retention time tolerance = 0.2 min (intra‐ and inter‐sample; Schmid *et al*., 2023).

To correct for analytical variation, duplicate extractions from a bulk aspen standard were included every 12 samples. Between‐batch variation was corrected by scaling concentrations using the ratio of the overall mean of the first two process standard injections to the batch‐specific mean. Within‐batch drift was corrected by fitting a linear model to process standard concentrations as a function of run order and applying the inverse correction to all samples.

SPG concentrations were calculated using standard curves and converted to percent dry weight as follows:

$$\begin{array}{l} \%\mathrm{DW}=\text{extraction}\;\mathrm{vol}.\times \text{dilution factor}\times 1.09\times 100\\ \;\times \frac{\text{sample concentration}}{\text{sample start weight}\left(g\right)\times 1000} \end{array}$$

where 1.09 is an empirically determined correction factor for extraction efficiency.

#### CT extraction and quantification

CTs were extracted in 70% acetone containing 0.01% ascorbic acid and quantified using the acid‐butanol colorimetric assay (Porter *et al*., 1985). Extracts were mixed with 95 : 5 butanol : HCl containing ferric ammonium sulfate and incubated at 95°C for 40 min. Absorbance was measured at 550 nm. Procyanidin B2 (≥ 90% purity; Sigma‐Aldrich, Cat. No. 42157) was used as the analytical standard.

#### Interannual change in chemistry

Interannual changes in SPGs and CTs were calculated by pairing samples across the years 2022 and 2023 and subtracting 2022 values from 2023 values for individuals with measurements in both years (*n* = 259).

### Tree growth and canopy damage

Relative growth rates (RGR) were calculated from annual stem diameter increments as described in Fickle *et al*. (2026). Approximately half of the trees in the common garden (*n* = 143) were harvested in June 2024, dried, and processed for high‐resolution stem cross‐sections. Annual ring boundaries were identified from scanned, polished stem disks and measured in ImageJ. For each individual, annual stem diameters were extracted for successive growth years from 2020 to 2024, and RGR was calculated as the relative change in stem diameter between the beginning and end of each growing season. Analyses in this study focused on RGR for the 2022–2023 growth year.

Canopy damage was scored in early September of each year based on a visual assessment of leaf condition across the entire canopy. This timing was chosen to capture the cumulative effects of drought stress while minimizing confounding effects of seasonal senescence. Leaf tissue showing chlorosis, necrosis, or complete loss (‘missingness’) was classified as damaged. For each tree, the proportion of canopy damaged was recorded as the proportion of canopy leaves affected.

### Microbial community characterization

#### Sampling and processing

Microbial community sampling was conducted concurrently with chemical sampling. To minimize diel variation in microbiome composition, leaves were collected in the morning (7:00 h–10:00 h) over 3–4 consecutive days. Equal numbers of samples were randomly selected from each plot on each day. Single leaves were clipped from mid‐canopy height into sterile tubes. As with chemical sampling, leaves were selected to be representative of each individual and were not chosen based on visible disease symptoms. Samples were washed in the field with 4–5 ml of 0.1% Tween‐20 solution, rinsed thoroughly with sterile deionized water, flash‐frozen on dry ice, and stored at −80°C.

At field sites, sampling occurred from late July to early September 2022 and over five consecutive days in mid‐July 2023. For each tree, three to four branches were collected using a 20‐gauge shotgun, and four leaves per branch were clipped into sterile tubes, washed, and stored as described above.

#### DNA extraction

DNA and RNA were co‐extracted from whole frozen leaves using a modified CTAB‐based protocol (Chang *et al*., 1993; Sasi *et al*., 2023; Lundberg *et al*., 2025). Although RNA sequencing data are not included in this analysis, the co‐extraction procedure is described here because it influenced the DNA extraction method. Leaves were ground in liquid nitrogen using sterile foil‐lined mortars and pestles and transferred to 2‐ml microcentrifuge tubes. Cell lysis was performed in CTAB buffer (100 mM Tris–HCl pH 8.0, 1.4 M NaCl, 20 mM EDTA, 2% CTAB, 2% polyvinylpyrrolidone K30, 2% β‐mercaptoethanol, 100 μg ml^−1^ Proteinase K) at 65°C for 60 min.

Nucleic acids were extracted using 24 : 1 chloroform:isoamyl alcohol and purified through two rounds of extraction. The aqueous phase was split, with 250 μl reserved for RNA extraction. DNA was precipitated with NaCl, potassium acetate, and isopropanol, incubated at −20°C overnight, centrifuged at 3200 ***g*** for 45 min, washed with 80% ethanol, dried, and resuspended in nuclease‐free water.

#### Amplicon sequencing

The ITS1 region was amplified using a two‐step PCR protocol with frameshifting primers following Regalado *et al*. (2020) and Lundberg *et al*. (2013), excluding blocking oligos. Primer sequences are provided in Table S1.

First‐round PCR reactions (60 μl total volume) contained ThermoPol buffer (1×; New Englad Biolabs, Ipswich, MA, USA), forward and reverse primers (0.08 μM), dNTPs (0.2 mM), Taq DNA polymerase, and template DNA (5 μl). Reactions were split across three plates and run in triplicate under the following conditions: 94°C for 2 min; 10 cycles of 94°C for 30 s, 55°C for 30 s, 72°C for 30 s; and 72°C for 3 min.

Amplified DNA was pooled and cleaned using 1 : 1 SPRI beads before a second PCR. Second‐round PCR reactions (25 μl) used Q5 polymerase with 1× Q5 buffer, 0.48 mM dNTPs, 0.5 μM forward primers and 0.25 μM barcoded reverse primers. Thermal cycling conditions were: 94°C for 1 min; 25 cycles of 94°C for 20 s, 60°C for 30 s, 72°C for 30 s; and a final extension at 72°C for 2 min.

Amplicon size (*c*. 400–450 bp) and adapter incorporation were verified by agarose gel electrophoresis. Amplicons were pooled by plate, cleaned using a 1 : 1.2 SPRI bead ratio, eluted, concentrated 10× at 40°C via vacuum centrifugation, and size‐selected (300–750 bp) using a BluePippin system (1.5% gel). Libraries were sequenced on an Illumina MiSeq using a v.3 600‐cycle kit.

#### Sequence processing and filtering

Reads were demultiplexed and adapters trimmed using custom scripts (Karasov, 2023). Reads were filtered and denoised without merging using DADA2 (parameters: maxN = 0, maxEE = 2, truncQ = 2, minLen = 50; Callahan *et al*., 2016).

Taxonomic assignment was performed on forward reads using the UNITE database v.9.0 general Fasta release (Abarenkov *et al*., 2024). ASVs were retained if classified as fungi at the family level or lower. Samples with fewer than 500 reads and ASVs detected in fewer than three samples were excluded before analysis. No rarefaction was performed, consistent with recommendations underlying the phyloseq framework, because rarefaction discards valid observations and can reduce precision and statistical power (McMurdie & Holmes, 2013). Alpha diversity metrics were calculated from the filtered dataset, whereas Bray–Curtis dissimilarities and subsequent PERMANOVA and dbRDA analyses were performed using relative abundance data. Differential abundance analyses were conducted using unrarefied count data.

#### Community classification and analysis framework

Microbial community analyses were initially conducted on the full dataset to characterize overall structure. Subsequent analyses focused on pathogen‐capable taxa to evaluate associations with drought and host chemistry. Genera were classified using the FungalTraits database (Põlme *et al*., 2020), supplemented by manual curation. The term ‘pathogen‐capable’ refers to taxa classified as plant pathogens in FungalTraits, including opportunistic pathogens.

### Statistical analyses

All analyses were conducted in R (v.4.5.2).

#### Effects of drought on foliar chemistry

Bray–Curtis dissimilarities were calculated from the full table of UPLC results normalized by sample weight using vegdist (vegan v.2.6.10). Principal coordinates analysis (PCoA) was performed using pcoa (ape v.5.8.1; Paradis, Claude & Strimmer, 2004), and treatment groups were compared using permanova (adonis2, vegan; Oksanen *et al*., 2025).

To evaluate drought effects on foliar chemistry, we fit linear mixed‐effects models using lme4 (v.1.1.36; Bates *et al*., 2015). Models included prior‐year treatment as a fixed effect and genotype as a random intercept:

$$y_{\mathrm{ij}}=\beta_{0}+\beta_{1}{\text{Treatment}}_{j}+b_{\text{genotype}\left[i\right]}+\varepsilon_{\mathrm{ij}}$$

where $y_{\mathrm{ij}}$ is the metabolite concentration for individual i in treatment group j, ${\text{Treatment}}_{j}$ is drought treatment group, $b_{\text{genotype}\left[i\right]}\sim N\left(0,\sigma_{\text{genotype}}^{2}\right)$ is the random intercept for genotype, and $\varepsilon_{\mathrm{ij}}\sim N\left(0,\sigma^{2}\right)$ is the residual error.

Separate models were fit for individual SPGs, total SPGs, CTs, and interannual changes in these traits. Interaction terms between genotype and treatment were not included because genotypes were not fully replicated across treatments, resulting in singular model fits and preventing reliable estimation of interaction effects.

Model assumptions were evaluated using *Q*–*Q* plots and residual‐fitted plots. Minor deviations from normality were observed but did not affect inference. Homoscedasticity and random effect distributions were acceptable.

Significance of fixed effects was evaluated using Type III ANOVA (car v.3.1.3). *Post hoc* comparisons were conducted usingemmeans (v.1.10.7) with Tukey correction (Lenth, 2025).

Variance explained was calculated using the partr2 package (v.0.9.2; Stoffel *et al*., 2021). Both Marginal and Conditional *R* ^2^ were calculated for each model. Marginal *R* ^2^ was used to estimate fixed effects (treatment), and the difference between conditional *R* ^2^ and marginal *R* ^2^ isolated the variance explained by genotype.

#### Associations between chemistry and performance

Growth (RGR) was analyzed using linear models, including changes in foliar chemistry (ΔSPGs, ΔCTs, Δtotal phenolics) and drought treatment variables. Interaction terms were included where biologically motivated.

Model assumptions were evaluated using *Q*–*Q* plots, residual‐fitted plots, and Breusch–Pagan tests. All models met assumptions, with minor deviations in upper tails. Heteroskedasticity‐consistent (HC3) SE were reported. A sensitivity analysis excluding a single extreme SPG value was also used to test the robustness of results.

Effects on canopy damage (2023) were analyzed using beta regression implemented in glmmtmb (v.1.1.14; McGillycuddy *et al*., 2025). Models included foliar chemistry and drought treatment as fixed effects, with dispersion allowed to vary by treatment group.

Model diagnostics were conducted using dharma (v.0.4.7; Hartig, 2026), which indicated no evidence of overdispersion or zero inflation.

Candidate models included treatment‐only, chemistry‐only, additive, and interaction models. Model selection was conducted using likelihood ratio tests and AICc (aiccmodavg v.2.3.4; Mazerolle, 2023).

For additive models, marginal effects were estimated using marginaleffects (v.0.32.0; Arel‐Bundock *et al*., 2024). For interaction models, treatment‐specific slopes were evaluated using emtrends, and predictions were generated using estimated marginal means.

#### Fungal community composition

Fungal community composition was analyzed in R using phyloseq (v.1.54.0) and vegan (McMurdie & Holmes, 2013). Shannon diversity was calculated using estimate_richness. Diversity differences were evaluated using linear models with site as a predictor.

Bray–Curtis dissimilarities were calculated using vegdist, and dbRDA was conducted using capscale (phyloseq). Models included treatment group, SPGs, and CTs as predictors, with garden block included as a conditioning variable. Significance was assessed using permutation tests (anova.cca, 999 permutations), and collinearity was evaluated using variance inflation factors (vif.cca).

#### Modeling pathogen‐capable taxa

The proportion of pathogen reads per sample was modeled using beta‐binomial GLMMs implemented in glmmTMB:

$$\text{logit}\left(p_{i}\right)=\beta_{0}+\beta_{1}{\mathrm{SPG}}_{i}+\beta_{2}{\text{Drought}}_{i}+b_{\text{block}\left[i\right]}$$

where $p_{i}$ is the proportion of pathogen reads, ${\mathrm{SPG}}_{i}$ is scaled total SPG concentration, ${\text{Drought}}_{i}$ is current‐year drought treatment, and $b_{\text{block}\left[i\right]}\sim N\left(0,\sigma_{\text{block}}^{2}\right)$ represents a random intercept for garden block.

Model diagnostics using DHARMa indicated no evidence of overdispersion or zero inflation.

Genus‐level responses to drought were modeled using beta‐binomial GLMMs for each genus:

$$\text{logit}\left(p_{\mathrm{ij}}\right)=\beta_{0}+\beta_{2}{\text{Drought}}_{i}+b_{\text{block}\left[i\right]}$$

where $p_{\mathrm{ij}}$ is the genus‐level relative abundance of genus i in sample j, ${\text{Drought}}_{i}$ is current‐year drought treatment, and $b_{\text{block}\left[i\right]}\sim N\left(0,\sigma_{\text{block}}^{2}\right)$ represents a random intercept for garden block.

Significant genera (*P* < 0.05) were identified based on model coefficients and compared with observed changes in relative abundance.

To compare experimental and field responses, genus‐level effect sizes were calculated as log‐odds contrasts and compared using variance‐weighted linear models, with weights defined as the inverse of the combined variance of the drought and field estimates:

$$w=\frac{1}{{\mathrm{SE}}_{\text{drought}}^{2}+{\mathrm{SE}}_{\text{field}}^{2}}$$

where w is the weight applied to each observation. Analyses were restricted to genera showing significant drought responses in the common garden.

#### Associations between taxa and host chemistry

For drought‐responsive genera, associations with host chemistry were evaluated using genus‐specific beta‐binomial GLMMs, including chemistry and drought as fixed effects and block as a random effect:

$$\text{logit}\left(p_{\mathrm{ij}}\right)=\beta_{0}+\beta_{1}{\text{Chem}}_{i}+\beta_{2}{\text{Drought}}_{i}+b_{\text{block}\left[i\right]}$$

where $p_{\mathrm{ij}}$ is the proportion of reads assigned to genus i in sample j, ${\text{Chem}}_{i}$ is the scaled measure of a single host chemistry class (either total SPGs or CTs), ${\text{Drought}}_{i}$ is current‐year drought treatment and $b_{\text{block}\left[i\right]}\sim N\left(0,\sigma_{\text{block}}^{2}\right)$ is a random intercept for garden block.

To evaluate whether Cladosporium enrichment was associated with declining canopy condition, we modeled Cladosporium relative abundance as a function of the proportion of canopy damaged while accounting for drought treatment and block structure using a beta‐binomial generalized linear mixed model:

$$\text{logit}\left(p_{i}\right)=\beta_{0}+\beta_{1}{\text{Canopy}}_{i}+\beta_{2}{\text{Drought}}_{i}+b_{\text{block}\left[i\right]}$$

where $p_{i}$ is the relative abundance of *Cladosporium* in sample i, ${\text{Canopy}}_{i}$ is the proportion of canopy damaged, ${\text{Drought}}_{i}$ is current‐year drought treatment and $b_{\text{block}\left[i\right]}\sim N\left(0,\sigma_{\text{block}}^{2}\right)$ is a random intercept for garden block.

#### ASV‐level differential abundance

Differential abundance was assessed using deseq2 (v.1.46.0; Love *et al*., 2014) with likelihood ratio tests and garden block included as a covariate. Dispersions were estimated using DESeq2's empirical Bayes approach, and dispersion‐mean relationships were visually inspected. Analyses focused on the strongest treatment contrast (CCC vs DDD), and *P*‐values were adjusted using the Benjamini–Hochberg (BH) method.

To facilitate ecological interpretation, FungalTraits genus‐level classifications were used to filter results to ASVs assigned to pathogen‐capable genera. This subset was selected to enable more interpretable comparisons of taxa likely to respond to host stress, rather than to exclude other ecologically relevant groups.

Because DESeq2 operates on count data and does not explicitly model proportional responses, ASV‐level results were compared with genus‐level and community‐level responses estimated using beta‐binomial GLMMs applied to focal treatment contrasts. Concordance between approaches was evaluated based on directional agreement of effect estimates (i.e. concordance in the sign of log_2_ fold changes and GLMM coefficients) and Spearman rank correlation of effect sizes.

## Results

### Prior‐year drought affects production of SPGs and CTs

We first asked whether foliar chemical profiles differed among drought treatments and whether this variation was associated with the focal SPG class. In 2022, PCoA1 of the UPLC data captured 67.8% of variation, with most contributors corresponding to peaks for the four major SPGs: salicin, salicortin, tremulacin, and tremuloidin (Fig. S1a). PCoA1 from 2023 (68.1% of variance) was also dominated by SPGs (Fig. S1b). While there was no significant separation between treatment groups in 2022, in 2023, trees experiencing two or three consecutive years of drought (CDD, DDD) separated from those with alternating drought and normal watering (DCD, CCC; *P* = 0.002 vs CCC, 0.024 and 0.002 vs DCD for CDD and DDD, respectively). DCD also separated from CCC (*P* = 0.024). PC2 described an additional 8.4% (2022) and 6.7% (2023) of variance and was associated with larger, more polar compounds that eluted early in the gradient.

Having observed separation of treatment groups along the axis describing variation in SPGs, we next explicitly tested whether current or prior‐year drought treatment was associated with changes in allocation to SPGs. Linear mixed‐effect models controlling for effects of genotype indicated that there was no significant difference in total SPGs between trees experiencing current‐year drought and those with normal watering in 2022 (*P* = 0.3417; Fig. 2a). Trees that experienced drought the prior year had higher SPG content by 1.46% dry weight, an 11% increase over plants that were normally watered the prior year (*P* < 0.0001), corresponding with increases in salicortin and tremulacin in the drought–control‐ and drought–drought‐treated trees (Fig. S2). Prior‐year water availability explains 7.7% of variance in total SPGs between samples, compared with 14.5% explained by genotype (Table 1). CT concentrations, as measured by colorimetric acid‐butanol assays, did not differ between treatments in 2022 (Fig. 2c). There was marginal evidence for a treatment effect on CT concentrations in 2022 (χ^2^ = 7.55, *P* = 0.056), corresponding with a trend toward higher CTs following a single year of drought (CDD vs CCC, *P* = 0.063). However, this pattern was not maintained under prolonged or repeated drought treatments.

**Fig. 2:**
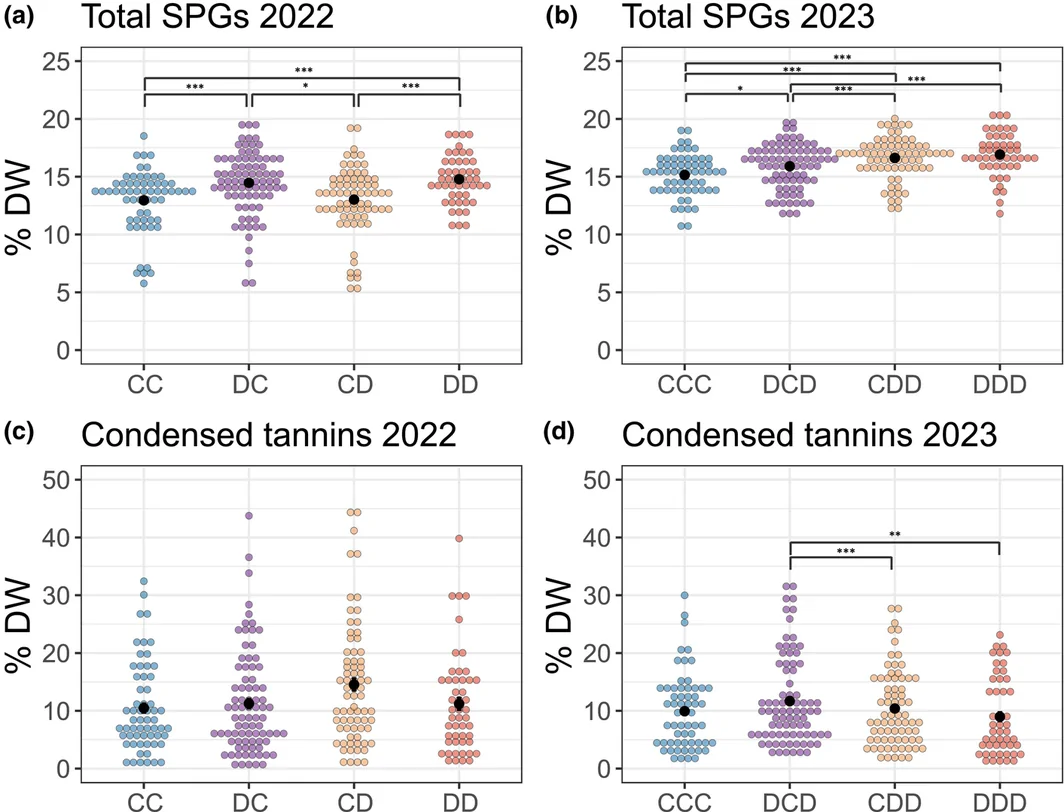
Drought memory shapes aspen (*Populus tremuloides*) allocation to foliar defensive chemistry. Percent dry weight (% DW) allocated to salicinoid phenolic glycosides (SPGs) was higher in *P. tremuloides* that experienced drought in the prior year in both 2022 (a) and 2023 (b). Condensed tannins (CTs) did not differ between treatment groups in 2022 (c) but were significantly lower in previously drought‐stressed trees in 2023 (d). Colored points represent individual samples; black points represent treatment mean; black lines show mean ± SE. Pairwise comparisons were performed using emmeans with Tukey‐adjusted *P*‐values: (***) < 0.001, (**) <0.01, (*) <0.05. Sample sizes: *n* ≤ 51 (CCC, DDD) or *n* ≤ 74 (CDD, DCD). CCC = control‐control‐control; CDD = control‐drought‐drought; DCD = drought‐control‐drought; DDD = drought‐drought‐drought.

**Table 1:** Variance explained (VE) in *Populus tremuloides* chemical defenses for treatment and genotype.

	Year	VE by genotype (%)	CI lower–upper	VE by treatment (%)	CI lower–upper
Salicin	2022	1.2	0.6–6.4	9.3	6.6–14.1
	2023	20.5	18.8–21.0	35.5	29.9–41.0
Salicortin	2022	15.3	12.3–16.7	8.9	3.3–13.0
	2023	38.1	32.0–39.6	10.7	6.9–17.1
Tremulacin	2022	17.3	11.1–17.5	6.3	3.2–13.6
	2023	56.5	44.8–60.8	5.5	4.6–8.2
Total SPGs	2022	14.5	9.3–19.2	7.7	5.1–11.8
	2023	46.3	28.8–48.0	10.9	8.4–15.0
CTs	2022	40.8	29.7–53.8	1.3	0.9–3.9
	2023	50.8	34.5–63.7	3.1	1.3–3.6

CI, 95% confidence interval; CTs, condensed tannins; SPGs, salicinoid phenolic glycosides; VE, variance explained.

### Prior‐year effects still dominate after prolonged drought

In 2023, total SPGs again differed primarily by prior‐year treatment, with an 8% increase in plants that had experienced drought the previous year compared with prior‐year controls (*P* < 0.0001; Fig. 2b). DCD plots were also significantly higher than controls (5%, *P* = 0.0173) despite sharing the same prior‐year treatment, a difference associated with significant increases in both salicin (26%, *P* < 0.0001) and tremulacin (4%, *P* = 0.0254; Fig. S2). It cannot be determined from the data whether this is due to the drought in 2021 or 2023 as these are correlated in the treatments. No significant differences were observed between the 2‐ and 3‐yr drought groups. Both genotype and treatment explained more variance in 2023 (46.3 and 10.9%, respectively), with treatment effects strongest for salicin and salicortin (Table 1). CTs in 2023 were significantly higher in the alternating drought treatment than in the 2‐ (31%, *P* = 0.0006) and 3‐yr (32%, *P* = 0.0011) consecutive drought plots (Fig. 2d). As in 2022, genotype explained more variation than treatment for all chemical defenses except salicin.

### Evidence for a shift in allocation between SPGs and CTs following drought

Comparisons among treatments revealed contrasting allocation patterns between SPGs and CTs in plots that switched treatments between 2021 and 2022. Between 2022 and 2023, all groups showed modest SPG increases, but the increase in CDD was 2.93‐fold greater than in DCD (*P* < 0.0001; Figs 3a, S3). By contrast, CT concentrations increased in DCD but decreased in CDD by a mean of 6.095% dry weight, with the year‐to‐year change differing by a mean of 6.77% dry weight between the groups (*P* = 0.0014; Figs 3b, S3, S4).

**Fig. 3:**
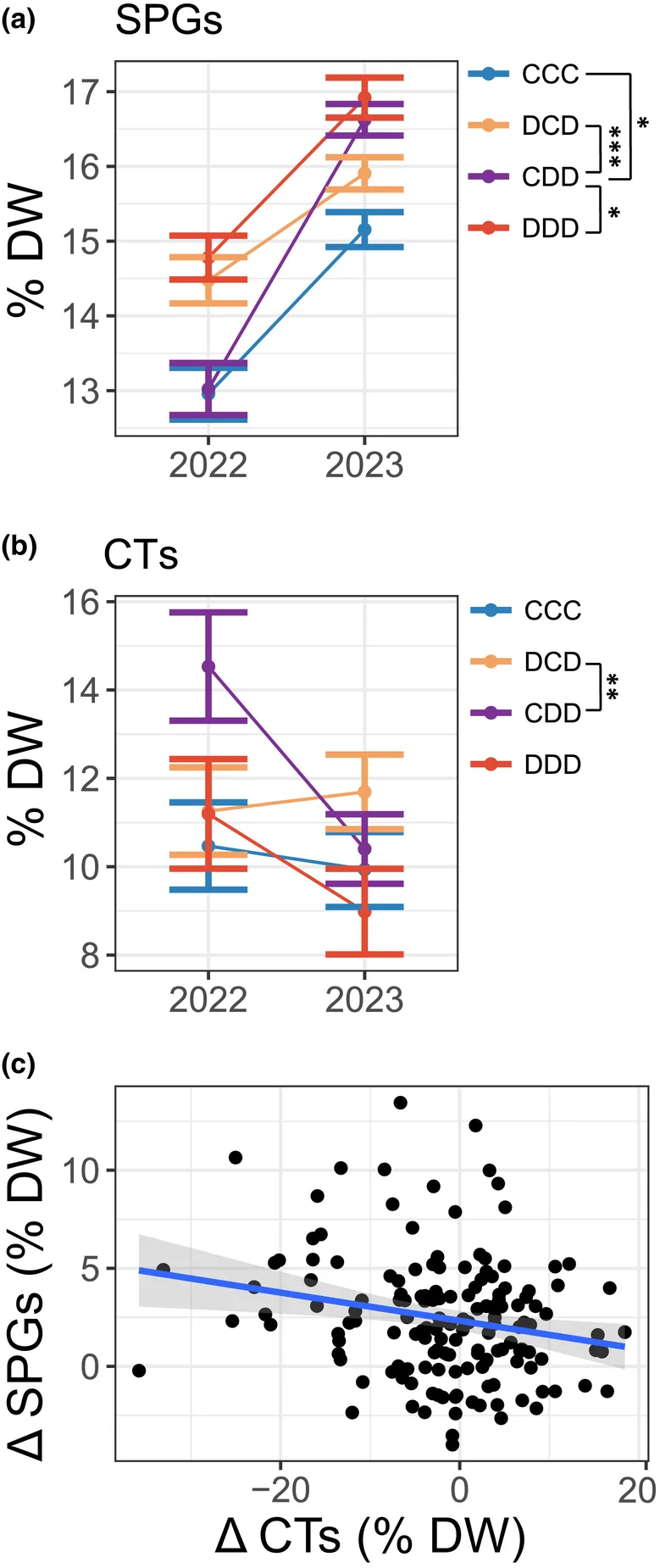
Drought memory in *Populus tremuloides* is associated with opposing shifts in salicinoid phenolic glycosides (SPGs) and CTs. Mean total percent dry weight (% DW) by treatment group for SPGs (a) and CTs (b) in 2022 and 2023; points show mean ± SE. Asterisks to the right of panels (a, b) indicate significant differences among treatment groups based on *post hoc* comparisons of within‐sample changes from 2022 to 2023 (estimated marginal means with Tukey‐adjusted *P*‐values; *, *P* < 0.05; **, *P* < 0.01; ***, *P* < 0.001). (c) The change in SPGs from 2022 to 2023 is inversely correlated with the change in CTs in the CDD and DCD treatment groups (slope = −0.07181, adjusted *R*² = 0.03996, *P* < 0.01). Shaded ribbon indicates 95% confidence interval. Sample sizes: *n* ≥ 51 (CCC, DDD) and *n* ≥ 74 (CDD, DCD). CCC = control‐control‐control; CDD = control‐drought‐drought; DCD = drought‐control‐drought; DDD = drought‐drought‐drought.

Across individuals, total SPG content and CT content were negatively correlated (slope = −1.16, adjusted *R* ^2^ = 0.1084, *P* < 0.0001). To determine whether individual trees were shifting their allocations between these two compound classes, we tested for correlation between year‐to‐year changes in SPGs and CTs within the groups that switched treatments between 2021 and 2022 (CDD and DCD). Changes were negatively correlated (slope = −0.07181, adjusted *R* ^2^ = 0.03996, *P* = 0.0074; Fig. 3c), consistent with an allocation trade‐off between SPGs and CTs due to drought induction.

### Associations between SPGs and growth and canopy condition

Having observed drought‐associated shifts in foliar chemistry, we next asked whether these changes were associated with differences in aspen performance, testing whether variation in foliar chemistry corresponded to differences in growth and leaf canopy condition. Growth differed significantly among drought treatments (Fig. 4a–c; Table 2). Changes in SPG allocation were positively associated with RGR, indicating that increased investment in SPGs was not accompanied by reduced growth (Fig. 4a). Exclusion of the extreme observation reduced statistical support for this relationship, although the direction and magnitude of the estimated effect were largely unchanged. There was no statistically significant association between CTs or total phenolics and RGR (Fig. 4b,c).

**Fig. 4:**
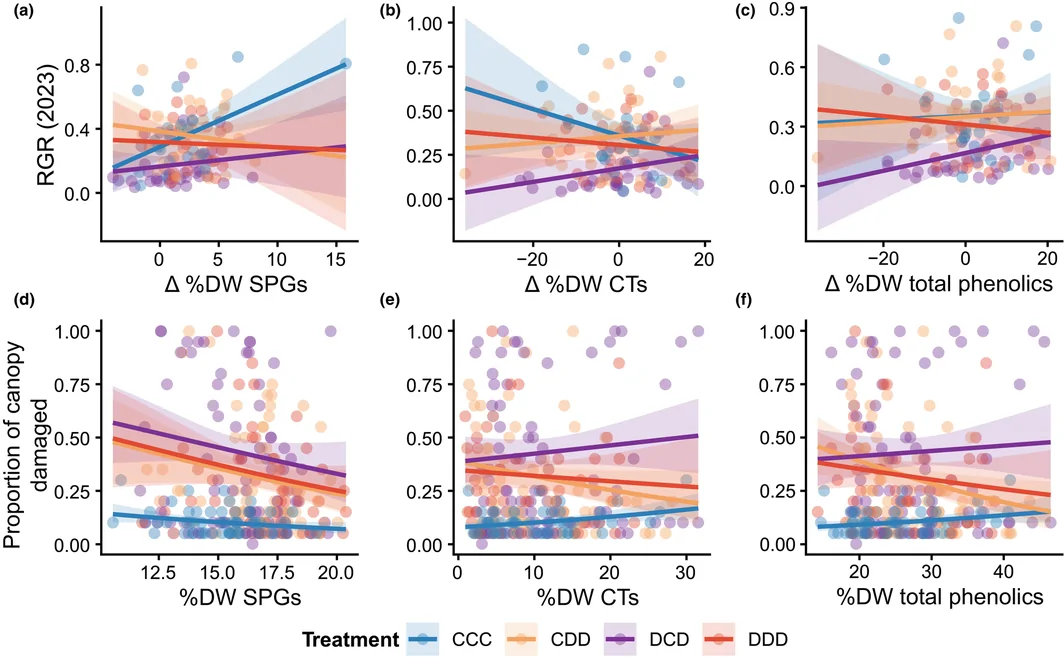
Drought‐associated changes in foliar chemistry track context‐dependent variation in aspen (*Populus tremuloides*) performance. (a–c) Relative growth rate (RGR, 2023) as a function of (a) salicinoid phenolic glycosides (SPGs), (b) condensed tannins (CTs), and (c) total phenolic chemistry. (d–f) Proportion of canopy damaged (2023) as a function of (d) SPGs, (e) CTs, and (f) total phenolic chemistry. Points represent individual trees. Lines show treatment‐specific model predictions from beta regression models, back‐transformed to the response scale and expressed as the predicted proportion of canopy damaged; shaded ribbons indicate 95% confidence intervals. Statistics and significance reported in Table 2. SPGs were positively associated with growth, although support for this relationship was sensitive to a single extreme observation and varied with prior‐year drought conditions (a). There was no statistically significant association between CTs or total phenolics and growth (b, c). SPGs were associated with reduced canopy damage (d), whereas relationships between canopy damage and both CTs and total phenolics depended on drought history, with directionally opposing patterns among treatments and weak or nonsignificant effects in some cases (e, f). CCC = control‐control‐control; CDD = control‐drought‐drought; DCD = drought‐control‐drought; DDD = drought‐drought‐drought.

**Table 2:** Associations between foliar chemistry and drought treatment with *Populus tremuloides* growth and canopy damage.

Relative growth rate (RGR)						
ΔSPGs × treatment						
Type III ANOVA	df	*F* value		*P*‐value		
		w/outlier	w/out outlier	w/outlier	w/out outlier	
~ΔSPGs	1	9.89	0.676	0.002	0.413	
~treatment	3	4.79	4.72	0.003	0.004	
~ΔSPGs × treatment	3	2.41	1.16	0.071	0.327	
**Wald tests of model coefficients**	**Estimate**	**SE**	***P*‐value**			
ΔSPGs	0.033	0.01	0.001			
ΔSPGs × CDD	−0.043	0.021	0.044			
ΔCTs × treatment						
**Type III ANOVA**	**df**	***F* value**	***P*‐value**			
~treatment	3	7.11	< 0.001			
**ΔTotal phenolics × treatment**						
**Type III ANOVA**	**df**	***F* value**	***P*‐value**			
~treatment	3	7.035	< 0.001			
**ΔSPGs × prior‐year drought treatment**						
**Type III ANOVA**	**df**	***F* value**		***P*‐value**		
		**w/outlier**	**w/out outlier**	**w/outlier**	**w/out outlier**	
~prior‐year drought	1	8.236	7.24	0.005	0.008	
~ΔSPGs × prior‐year drought	1	4.462	1.77	0.037	0.187	
**Wald tests of model coefficients**	**Estimate**	**SE**	**P‐value**			
Prior‐year drought	−0.072	0.027	0.009			
**Estimated marginal trends**	**Estimate**	**SE**	**df**	***P*‐value**		
Control–drought	0.0297	0.014	106	0.037		
**Canopy damage**						
**Model selection and interaction tests**	**Comparison**		**ΔAIC**	**χ** ^**2**^	**df**	***P*‐value**
SPGs	Additive vs treatment‐only		−8.95	10.94	1	< 0.001
CTs	Interaction vs additive		−2.66	8.66	3	0.034
Total phenolics	Interaction vs additive		−4.29	10.28	3	0.016
**Estimated effects on canopy damage**	**Treatment**	**Effect (β or marginal effect ± SE)**			**95% CI**	***P*‐value**
SPGs	All (additive)	−1.8% per 1% DW			[−2.9, −0.8]	< 0.001
CTs	CCC	0.027 ± 0.010			—	0.0099
	CDD	−0.031 ± 0.018			—	0.089
Total phenolics	CCC	0.022 ± 0.011			—	0.055
	CDD	−0.048 ± 0.020			—	0.019

AIC, Akaike information criterion; CI, confidence interval; CTs, condensed tannins; df, degrees of freedom; SPGs, salicinoid phenolic glycosides.

There was some evidence that the relationship between SPG allocation and growth differed by drought history, although support for this pattern depended on the inclusion of the extreme observation (Fig. S5). In trees that experienced favorable water conditions in the previous year, increases in SPG allocation were positively associated with RGR, whereas this relationship was neutral to weakly negative following prior‐year drought. Simple slope analyses supported this pattern, indicating a positive association in trees without prior‐year drought exposure, but no detectable relationship in trees that experienced prior‐year drought.

We next tested whether foliar chemistry was associated with canopy damage. In 2023, the proportion of canopy damaged ranged from 0 to 1 (median = 0.15) and was elevated across all drought‐exposed treatments (CDD, DCD, and DDD) relative to CCC, with the strongest increase observed in DCD (Fickle *et al*., 2026). Total SPG concentration was associated with reduced canopy damage, with a 1 percent dry weight increase in SPGs corresponding to a 1.8% decrease in expected damage (Fig. 4d). By contrast, associations between canopy damage and both CTs and total phenolics depended on drought history (Fig. 4e,f). In continuously watered trees (CCC), higher concentrations were associated with increased canopy damage, whereas in trees that experienced drought in the previous year (CDD), higher concentrations were associated with reduced canopy damage. Relationships were weak or nonsignificant in the remaining treatments, indicating that the effects of phenolic investment on canopy damage varied with drought history.

### Potential pathogens show association with prior‐year treatment

To characterize baseline fungal community composition and assess whether the common garden reflects field‐associated communities, we surveyed foliar fungal communities at peak drought. Our survey of foliar fungal communities at peak drought identified 6467 unique ASVs in 2022 and 8290 ASVs in 2023 after filtering for family‐level assignments, with *Cladosporium* and *Comoclathris* the most abundant taxa across treatments – consistent with previous *Populus* phyllosphere surveys (Figs S6, S7). We compared community composition in the common garden in 2022 with that of the field sites and found that the garden composition broadly corresponded to composition in the field, with no significant differences in incidence of *Cladosporium*, *Didymella*, *Dothidotthia*, or *Fusarium* (BH‐adjusted *P* > 0.01). Garden samples had higher *Comoclathris* abundance (BH‐adjusted *P* < 0.0001) and lower overall diversity than field samples (Shannon diversity difference = −0.456, *P* < 0.0001), suggesting that the common garden is a reasonable but slightly less diverse representation of microbial communities associated with these genotypes.

Having established that the common garden broadly reflects field‐associated communities, we next asked whether drought and host chemistry were associated with shifts in the fungal community. Distance‐based redundancy analysis (dbRDA) showed significant differences in fungal community composition between treatment groups (Fig. S8). The full model including treatment group, total SPGs and CTs explained a modest but significant proportion of variation in community composition (*R* ^2^ = 0.039, *P* = 0.001). The first two constrained axes explained 44.4% and 26.6% of constrained variation, respectively, and were both significant (CAP1: *P* = 0.001, CAP2: *P* = 0.035), indicating that community responses to drought and host chemistry were structured along more than one axis of constrained variation. Drought‐treated groups largely overlapped with one another but were shifted relative to controls along the primary axis (*P* = 0.001). In addition, SPGs were significantly associated with community structure (*P* = 0.007), whereas CTs showed no detectable effect (*P* = 0.636).

To determine whether these community‐wide shifts reflected changes in ecologically relevant community characteristics, we next used FungalTraits to classify fungal ASVs as pathogen or nonpathogen and tested whether the relative abundance of pathogen‐capable taxa was associated with drought and SPGs. Given the strong overlap among drought treatment groups in the dbRDA, we simplified treatment to a current‐year drought vs control contrast and compared candidate models, including drought, SPGs, and their interaction. The best‐supported model included additive effects of drought and SPGs, with no evidence for an interaction (ΔAIC ≈ 0.1; LRT *P* = 0.145). Pathogen‐capable fungi were more abundant under current‐year drought (β ≈ 0.24 ± 0.09 SE, *P* = 0.008) and decreased with increasing SPG concentrations (β ≈ −0.08 ± 0.04 SE, *P* = 0.027; Fig. 5a).

**Fig. 5:**
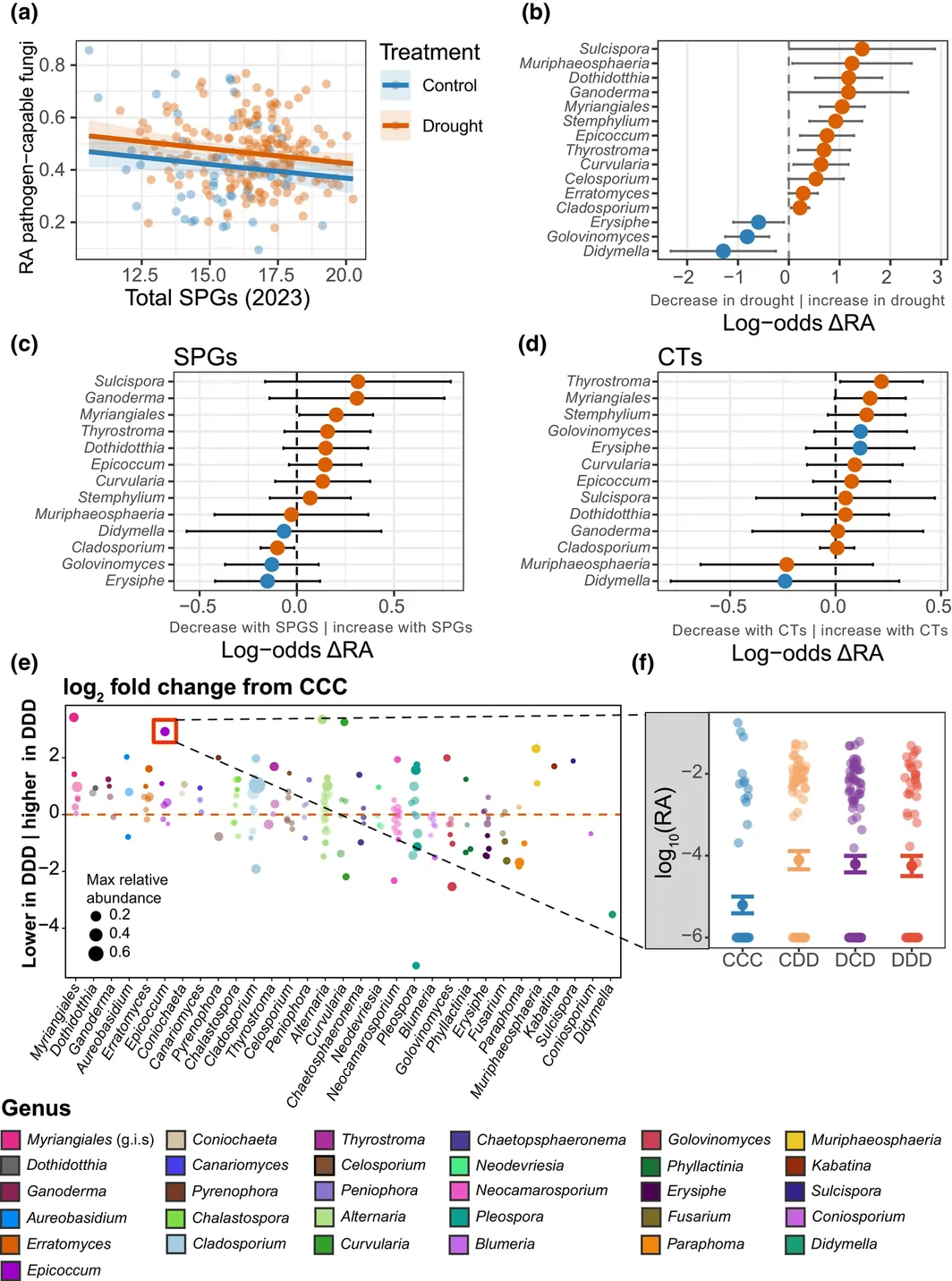
Responses of pathogen‐capable fungi to drought and foliar chemistry vary across taxa (a) Relative abundance (RA) of pathogen‐capable fungi identified by IT sequencing from *Populus tremuloides* leaves as a function of total salicinoid phenolic glycoside (SPG) concentration (2023), shown for control and drought treatments. Lines indicate model fits with 95% confidence intervals. (b) Genus‐level changes in relative abundance under drought, expressed as log‐odds differences (ΔRA). Positive values indicate higher relative abundance in drought relative to control, and negative values indicate decreases. Points are colored by direction of change (higher in drought vs control), with horizontal bars indicating 95% confidence intervals. (c, d) Genus‐level associations between relative abundance and (c) SPGs and (d) CTs, expressed as log‐odds ΔRA per 1% dry weight change in chemistry. Positive values indicate increasing relative abundance with higher chemical concentrations. Points are colored to match panel b (indicating whether each genus is more abundant in drought or control), with horizontal bars indicating 95% confidence intervals. (e) Log₂ fold change in relative abundance of individual genera in DDD (drought‐drought‐drought) relative to CCC (control‐control‐control). Points represent unique amplicon sequence variants (ASVs) within genera. Solid points are significant (Benjamini–Hochberg adjusted *P* < 0.05). Point size reflects maximum observed RA. (f) Distribution of RA (log₁₀‐transformed) for a representative ASV (*Epicoccum italicum*), shown across treatments; each point represents a single sample, with bars indicating mean ± SE. Across panels, drought was associated with increased relative abundance of several pathogen‐capable genera (b). Associations between SPGs and relative abundance were variable across taxa, with only a subset showing consistent directional responses (a, c). By contrast, relationships with CTs were generally weak and inconsistent (d), indicating taxon‐specific responses that depend on both compound class and drought context.

To determine which taxa contributed to the drought‐associated increase in pathogen‐capable fungi, we next modeled genus‐level responses to current‐year drought within the subset of taxa classified as pathogens. Responses were heterogeneous across genera, with multiple taxa showing significant increases or decreases in relative abundance under drought (Fig. 5b). Several genera exhibited positive responses to drought, including *Myriangiales* (β = 1.04, *P* < 0.001), *Dothidotthia* (β = 1.18, *P* < 0.001), and *Epicoccum* (β = 0.82, *P* = 0.002), while others such as *Erysiphe* (β = −0.71, *P* = 0.006) and *Didymella* (β = −1.29, *P* = 0.014) declined. These opposing responses indicate that the overall increase in pathogen‐capable fungi arises from shifts in a subset of responsive taxa rather than a uniform community‐wide response.

Despite this heterogeneity, changes in community composition were disproportionately driven by a small number of taxa. Cladosporium accounted for the largest proportional increase in pathogen‐capable fungi under drought, substantially exceeding other genera (Fig. S9), and showed a positive model‐estimated drought response (β = 0.22, *P* = 0.015). However, contributions were distributed across multiple taxa, indicating that drought‐driven shifts in pathogen abundance reflect both dominant and diffuse components of community change. Model‐estimated drought effects were generally consistent with observed proportional changes across genera, supporting the robustness of these patterns across analytical approaches.

Across genera, drought‐associated responses in the common garden were positively correlated with site‐level associations across field sites (Table S2). Genera that increased under experimental drought also tended to be more abundant at the driest site (UNCO) relative to the wettest site (UNT; variance‐weighted linear model: slope = 1.52 ± 0.48, *P* = 0.011, *R* ^2^ = 0.53).

Having identified the taxa driving drought‐associated shifts in pathogen abundance, we next asked whether these responses were associated with host chemical defenses. Specifically, we tested whether the relative abundance of drought‐responsive genera was related to concentrations of SPGs and CTs. Associations with SPGs varied across genera and were not uniformly suppressive (Fig. 5c). Notably, *Cladosporium*, the dominant contributor to the drought‐associated increase in pathogen‐capable fungi, showed a significant negative association with SPGs (β = −0.10, *P* = 0.023), consistent with the overall decline in pathogen abundance at higher SPG concentrations observed at the community level (Fig. 5a). By contrast, other drought‐responsive taxa showed neutral or positive associations with SPGs. For example, *Myriangiales*, which also increased under drought, exhibited a positive association with SPGs (β = 0.20, *P* = 0.037).

Associations with CTs were weaker and less consistent across taxa (Fig. 5d). Only a small number of genera showed significant relationships with CT concentrations, including *Thyrostroma*, which exhibited a positive association (β = 0.22, *P* = 0.029). However, these effects were not directionally consistent across taxa and did not correspond to a clear shift in pathogen abundance at the community level. Together, these results suggest that CTs play a more limited role than SPGs in structuring pathogen‐capable fungal communities.

While it was the largest contributor to the drought‐associated increase in pathogen‐capable fungi, Cladosporium is not a strictly pathogenic genus. Cladosporium includes taxa with endophytic, saprotrophic, and opportunistic lifestyles. Because associations between Cladosporium abundance and canopy damage could provide insight into its ecological role – for example, enrichment on damaged tissue might be consistent with saprotrophic growth or opportunistic pathogenicity, whereas enrichment on relatively healthy canopies could suggest alternative ecological roles – we tested whether Cladosporium abundance covaried with the proportion of canopy damaged. After accounting for treatment effects, Cladosporium relative abundance was not significantly associated with the proportion of canopy damaged (β = −0.043, *P* = 0.16).

While genus‐level analyses identified taxa associated with drought and host chemistry, these patterns assume that all ASVs within a genus respond similarly. To evaluate this assumption at finer taxonomic resolution, we examined ASV‐level differential abundance within pathogen‐capable genera using the strongest treatment contrast, comparing fully watered controls (CCC) with repeated drought (DDD).

ASV‐level responses in the CCC vs DDD comparison were often, but not universally, consistent within genera (Fig. 5e). In several cases (e.g. *Myriangiales*, and *Epicoccum*), most ASVs within a genus shifted in the same direction, supporting the genus‐level drought associations described above. However, other genera (e.g. *Alternaria*, and *Cladosporium*) contained ASVs with both positive and negative responses, indicating substantial within‐genus heterogeneity under the strongest drought contrast. Comparison of DESeq2 log_2_ fold changes with beta‐binomial mixed‐model estimates revealed strong concordance between methods. Effect directions agreed for 94% of ASVs, and effect sizes were positively correlated (Spearman's ρ = 0.70), indicating consistent patterns of taxon response to drought. Although fewer ASVs reached statistical significance in the mixed‐model framework, likely due to its more conservative structure, the high agreement between approaches supports the robustness of differential abundance results.

## Discussion

Specialized metabolites are key determinants of tree tolerance to biotic and abiotic stresses. Here, we show that foliar chemistry in aspen retains a memory of prior environmental conditions. SPG allocation, which is typically stable within a growing season and largely structured by genotype (Lindroth *et al*., 2002; Donaldson *et al*., 2006), increased following prior‐year drought but showed little response to current‐year drought. This pattern suggests that drought effects on SPGs are established before or during early leaf development and persist across growing seasons, such that a single drought event can leave a lasting chemical signature.

As has been previously found in aspen, genotype explained a substantial proportion of variation in phenolic chemistry across treatments. Although we did not explicitly model genotype‐by‐treatment interactions, prior work in this system indicates intraspecific variation in drought‐response traits across populations (Kerr *et al*., 2023). We also observed considerable heterogeneity in year‐to‐year changes in chemical allocation among individuals, particularly under alternating treatment regimes (Fig. S3), suggesting that genotypes may differ in both the magnitude and direction of chemical responses to drought history. Together, these patterns are consistent with genotype‐by‐environment interactions, even though they were not formally tested here, and suggest that host heterogeneity in chemical defenses may influence postdrought recovery and biotic interactions.

Drought has been associated with elevated feeding preference in some insect guilds (Anderegg *et al*., 2015; Gely *et al*., 2020; Eisenring *et al*., 2024). It is possible that drought‐mediated increases in herbivory contribute to the observed increases in SPG production. While herbivory was observed in the garden, it was not explicitly measured. However, the spatial intermixing and replication of our treatment plots reduce the likelihood that herbivory alone accounts for the observed pattern. Whether acting directly or indirectly, the environmental drivers of increased SPGs appear to be linked to drought.

CTs are typically more inducible than SPGs (Hemming & Lindroth, 1999; Lindroth *et al*., 2002; Donaldson & Lindroth, 2007). Consistent with this, we observed weak evidence for transient increases following a single year of drought, but CTs declined under multi‐year drought. This decline was unexpected given proposed roles in photoprotection and oxidative stress mitigation (Gourlay & Constabel, 2019; Gourlay *et al*., 2022), and suggests that CT induction may not be sustained under prolonged or repeated stress.

The divergent responses of these compound classes to prolonged drought raise the question of whether drought‐associated shifts in chemical allocation translate into differences in plant performance. We tested this by evaluating leaf damage as an indicator of canopy health. SPG concentrations were consistently associated with reduced leaf damage, whereas relationships with CTs and total phenolics depended on drought history, with higher concentrations associated with reduced damage under prior drought but increased damage under well‐watered conditions.

These results suggest that CT investment may be beneficial under drought conditions but that these benefits are context‐dependent, consistent with a scenario in which inducible defenses would be advantageous. One possibility is that allocation within the phenylpropanoid pathway is temporally structured: SPGs are largely determined early in leaf development and respond to prior environmental conditions, whereas CTs remain more inducible within a growing season. Under this framework, delayed increases in SPGs following drought may constrain subsequent CT induction.

Trade‐offs with chemical allocation are a central theme in chemical ecology. The trade‐off between SPGs and CTs has been consistently demonstrated in both surveys of variation within genotypes and through experimental manipulation of expression in the phenylpropanoid pathway (Donaldson *et al*., 2006; Boeckler *et al*., 2014; Cole *et al*., 2016; Rubert‐Nason & Lindroth, 2021; Lindroth *et al*., 2023). However, evidence for a trade‐off due to environmental induction is mixed. Here, we specifically tested whether drought‐induces shifts in allocation consistent with an SPG‐CT trade‐off. Here, drought was associated with increased SPGs and reduced CTs, consistent with an induced shift in allocation. However, we found no evidence for a trade‐off between phenolic investment and growth. Associations between SPGs and RGR were neutral to positive and varied with drought history, suggesting that allocation‐growth relationships are context‐dependent rather than constrained by a fixed trade‐off.

The observed increase in SPGs and decrease in CTs during drought parallels geographic variation in aspen, where western populations tend to exhibit higher SPGs and lower CTs compared with northern and Great Lakes populations (Lindroth *et al*., 2023). While these geographic differences likely reflect long‐term evolutionary and demographic processes rather than short‐term plastic responses, the similarity in direction suggests that drought may reinforce or contribute to these broader patterns. Cytotype variation may also contribute, as triploid genotypes—common in western populations—differ in phenolic allocation, particularly during stress (Eisenring *et al*., 2022). Diploid genotypes appear to exhibit stronger phenolic trade‐offs in these systems, suggesting that cytotype may shape allocation strategies under drought (DeRose *et al*., 2022). Because most genotypes in our common garden were triploid, an important next step will be to evaluate whether similar patterns hold across populations with greater cytotypic diversity. More broadly, integrating postdrought longitudinal measurements of chemical investment with performance across genotypes and environments will be critical for determining how alternative allocation strategies contribute to resilience.

A key implication of these shifts in foliar chemistry is their potential to influence biotic interactions. In line with this expectation, our results point to structured, taxon‐specific responses of foliar fungi to drought and host chemistry. Only a subset of taxa responded to drought, and associations with foliar chemistry were similarly variable, suggesting that drought legacies may extend to microbial communities, but not uniformly across taxa. Differences among closely related taxa, including within genera, further indicate that traits influencing host association may operate at finer taxonomic scales. While the effect sizes observed here were generally modest, the consistency of these patterns across taxa suggests that drought and host chemistry may act as selective filters on microbial communities, although the functional consequences of these shifts remain uncertain.

Importantly, many of the taxa identified as ‘pathogen‐capable’ encompass a range of ecological strategies, including saprotrophic and potentially beneficial interactions, and their roles may shift depending on host condition and environment. For example, *Epicoccum*, which we observed at higher abundance in drought (Fig. 5f), has been reported as both an emerging pathogen in some systems (e.g. leaf spot and seed rot diseases) and as a beneficial or biocontrol‐associated taxon in others, including potential roles in inducing host defenses, including drought‐induced shifts in phenolic chemistry (Del Frari *et al*., 2019; Falk De Avila *et al*., 2025; Rashad *et al*., 2025; George *et al*., 2026). Such functional flexibility complicates interpretation of taxonomic shifts and highlights the need to link community composition with functional outcomes.

Similarly, although *Cladosporium* was the largest contributor to the drought‐associated increase in pathogen‐capable fungi, its ecological role in this context remains uncertain. *Cladosporium* is not generally recognized as a major foliar pathogen of aspen, though it has been shown to cause disease in other plant hosts (Tyagi *et al*., 2023). Members of the genus are more commonly associated with endophytic and saprotrophic lifestyles, and several species have been reported as mycoparasites of Melampsora spp. that may contribute to suppression of rust disease in aspen (El‐Dawy *et al*., 2021). *Cladosporium* abundance was not significantly associated with the proportion of canopy damaged after accounting for treatment effects, suggesting that increases in *Cladosporium* should not be interpreted as direct evidence of elevated pathogen pressure or as a simple response to declining leaf condition.

The patterns we observed are further supported by overlap between taxa enriched under drought in the common garden and those more abundant in the driest field populations, suggesting that similar ecological filters may operate across experimental and natural settings, although differences in tree age and environmental history between the garden and field limit direct comparison (Table S2). For example, *Golovinomyces*, a genus associated with powdery mildew, was less abundant under drought and at the driest field site and tended to be associated with lower SPG concentrations, consistent with expectations for a pathogen that favors well‐watered hosts (Bradshaw *et al*., 2017; Qiu *et al*., 2018; Wiseman *et al*., 2021). By contrast, *Ganoderma* was more abundant under drought in the garden and present at high abundance in some samples at our driest field site. *Ganoderma* is considered a secondary pathogen of aspen, but has been observed to be more damaging to aspen suffering from drought (Worrall & Fairweather, 2009; Dudley *et al*., 2015; Kües *et al*., 2015). Similarly, members of the Myriangiales, many of which are identified as pathogens of leaves and meristems, were enriched under drought and at drier field sites and were associated with both higher SPGs and CTs suggesting they may be more tolerant of chemically defended hosts (Tsuneda *et al*., 2008; Fan *et al*., 2017; Barreto *et al*., 2024).

While these examples are illustrative and not intended to establish causal relationships, they highlight the potential for drought and host chemistry to act as selective filters on microbial communities in ways that depend on both taxonomic identity and ecological strategy. Understanding these interactions will require linking chemical traits, microbial functional traits, and host conditions across environmental gradients.

In deciduous trees like aspen, the physiological and molecular mechanisms behind drought stress memory remain poorly understood. In woody tissues, drought‐induced damage to fine roots and xylem can limit water and nutrient uptake long after the period of drought, reducing resource availability and potentially constraining how leaves allocate resources to metabolism. Alternatively, persistent changes in carbohydrate reserves due to reduced photosynthesis during drought or alterations to the regulation of specialized metabolite pathways could produce altered chemical profiles following drought stress. In other plant systems, drought‐induced epigenetic modifications, small RNAs, and transcriptional reprogramming have been associated with changes to stress tolerance following drought events, though few of these studies have been conducted in perennial plants or over multiple growing seasons (Auler *et al*., 2021; Sharma *et al*., 2022; Siddique *et al*., 2024). The paucity of documented examples in deciduous trees highlights a key gap in our understanding. Our results show a foliar chemical legacy of drought exposure, but further research is needed to ascertain whether the observed pattern is driven by active regulation or an indirect effect of structural damage to other plant tissues. It further remains to be determined how these changes interact with subsequent biotic and abiotic challenges and whether they are beneficial to postdrought recovery or likely to exacerbate the detrimental effects of drought.

In either case, these legacy effects may have far‐reaching ecological consequences. In aspen, severe multi‐year drought has been identified as the major driver of ‘Sudden Aspen Decline’ (SAD; Worrall *et al*., 2010, 2015). Canopy dieback and mortality are often followed by increased prevalence of opportunistic pathogens such as *Cytospora chrysosperma*, *Ganoderma applanatum*, and insect pests (Marchetti *et al*., 2011; Dudley *et al*., 2015). Our results suggest that drought‐induced shifts in chemical defenses may contribute to postdrought vulnerability in aspen by influencing stress tolerance and biotic interactions beyond the immediate period of drought. More broadly, climate change is predicted to intensify and prolong periods of drought in many regions (Dai, 2013; Cook *et al*., 2015). Similar dynamics have been observed in other systems; for example, drought constrains induction of terpenes and increases susceptibility of *Pinus edulis* to bark beetle attack (Gaylord *et al*., 2013). This raises the possibility of more frequent large‐scale stress‐associated pathogen or pest outbreaks in forest trees. Understanding how drought legacies in plant chemistry mediate biotic interactions will be critical for predicting forest recovery and informing management strategies in a rapidly changing climate.

## Competing interests

None declared.

## Author contributions

AMH, JCF, KLK, WRLA and TLK contributed to the project planning and experimental design. JCF and KLK conducted the physiological measurements. AMH, AMP, ES, MA and CF carried out the fieldwork and sample processing. AMH and HNC conducted UPLC experiments. AMH, DLF and RLL performed the metabolomics processing and analysis. AMH and TLK conducted the data analysis. AMH, RLL, WRLA and TLK contributed to writing the manuscript. WRLA and TLK contributed equally to this work.

## Disclaimer

The New Phytologist Foundation remains neutral with regard to jurisdictional claims in maps and in any institutional affiliations.

## Supporting information


**Fig. S1** Multivariate patterns of *Populus tremuloides* foliar chemistry across drought treatments.
**Fig. S2** Drought legacy effects on *Populus tremuloides* allocation to individual salicinoid phenolic glycosides.
**Fig. S3** Changes in allocation to *Populus tremuloides* defensive chemistry differ among drought treatments.
**Fig. S4** Drought legacy effects on individual salicinoid phenolic glycosides in *Populus tremuloides*.
**Fig. S5** Relationship between *Populus tremuloides* salicinoid phenolic glycoside allocation and relative growth rate depends on prior‐year drought treatment.
**Fig. S6** Relative abundance of dominant fungal genera across field sites in 2023.
**Fig. S7** Genus‐level composition of foliar fungal communities across drought treatments in the common garden.
**Fig. S8** Constrained ordination of foliar fungal communities from *Populus tremuloides* in the common garden.
**Fig. S9** Concordance between model‐estimated drought effects and observed changes in relative abundance of fungal genera in common garden aspen (*Populus tremuloides*).
**Methods S1** Common garden design and drought treatment.
**Table S1** ITS PCR Oligos (from Lundberg *et al*., 2013; Regalado *et al*., 2020).
**Table S2** Beta‐binomial GLMM results for taxa most associated with drought‐stressed *P. tremuloides* in the common garden with corresponding results from the comparison between the driest (Uncompahgre National Forest) and wettest (Uinta National Forest) sampled field sites.
Please note: Wiley is not responsible for the content or functionality of any Supporting Information supplied by the authors. Any queries (other than missing material) should be directed to the *New Phytologist* Central Office.

## Data Availability

Sequencing data are available on the SRA database, accession PRJNA1372733. Sample metadata and chemistry data are available through Dryad: doi: 10.5061/dryad.bzkh189qb. Scripts are available at https://github.com/AubreyHawks/aspen_chem_microbiome.
