# Supplementary material for: Functional memory of drought affects leaf chemical defenses and microbial interactions in aspen
Source: New Phytol. 2026 Sep 13;252(4):1413–31. doi: 10.1111/nph.71561 (PMC13669813; doi:10.1111/nph.71561)
Supplement: Supplementary file 1 [file NPH-252-1413-s001.pdf]

**New Phytologist Supporting Information**

**Article title:** Functional memory of drought affects leaf chemical defenses and microbial interactions in aspen

**Authors:** Aubrey M. Hawks, Jaycie C. Fickle, Kelly L. Kerr, Dale L. Forrister, Allison M. Perkins, Efthymia Symeonidi, Madelyn Allen, Cannon Fehrenbach, Hsiao-Nung Chen, Richard L. Lindroth, William R.L. Anderegg, Talia L. Karasov

**Article acceptance date:** 23 July 2026

## Methods S1. Common garden design and drought treatment

### Propagation and garden establishment

Clones were derived from field-collected rootstock in 2019. Rootstock was treated with 0.3% indole-3-butyric acid to promote rooting, then grown in greenhouse conditions prior to transplantation into the common garden in May 2020. Plants were grown in Metro Mix 900 soil prior to field establishment.

Propagation success varied among genotypes and source populations. Each source population contributed 4-8 genotypes, with individual genotypes represented by 3-22 ramets. Of the 29 genotypes established, 12 were represented by at least three replicates per treatment group. All genotypes were retained in statistical analyses. Full genotype and replication details are provided in Kerr et al. (2023).

### Drought treatment implementation

In 2021, all plants were watered to field capacity from mid-May to mid-June prior to treatment initiation. Mean plant height at treatment onset was 18.35 cm (range: 3–108.5 cm). Drought treatments were imposed by reducing irrigation to 50% and then 25% of control levels based on soil moisture measurements.

Treatment assignments were modified across years to generate variation in drought history. In 2022, two blocks per treatment retained their original assignments, while others were switched (control-to-drought: CDD; drought-to-control: DCD). In 2023, all non-control blocks were subjected to drought.

### Drought monitoring

Drought severity was monitored using weekly measurements of predawn (04:00–06:00) and midday (12:00–14:00) leaf water potential using a pressure chamber (Model 1505D, PMS Instrument Company). Measurements were taken on a subset of six plants per block. Additional details are provided in Fig. S1 and Tables S1–S2.

**Table S1. ITS PCR Oligos (from Lundberg et al, 2013 and Regalado et al., 2020)**

| | |
|---|---|
| **PCR1** | |
| **ITS1 Forward** | |
| AgITS1F1 | ACACTCTTTCCCTACACGACGCTCTTCCGATCTGGCTTGGTCATTTAGAGGAAGTAA |

| | |
|---|---|
| AgITS1F2 | ACACTCTTTCCCTACACGACGCTCTTCCGATCTTGGCTTGGTCATTTAGAGGAAGTAA |
| AgITS1F3 | ACACTCTTTCCCTACACGACGCTCTTCCGATCTCTGGCTTGGTCATTTAGAGGAAGTAA |
| AgITS1F4 | ACACTCTTTCCCTACACGACGCTCTTCCGATCTACTGGCTTGGTCATTTAGAGGAAGTAA |
| AgITS1F5 | ACACTCTTTCCCTACACGACGCTCTTCCGATCTGACTGGCTTGGTCATTTAGAGGAAGTAA |
| AgITS1F6 | ACACTCTTTCCCTACACGACGCTCTTCCGATCTTGACTGGCTTGGTCATTTAGAGGAAGTAA |
| **ITS1 Reverse** | |
| AgITS2R1 | GTGACTGGAGTTCAGACGTGTGCTCTTCCGATCTGGCTGCGTTCTTCATCGATGC |
| AgITS2R2 | GTGACTGGAGTTCAGACGTGTGCTCTTCCGATCTTCGGCTGCGTTCTTCATCGATGC |
| AgITS2R3 | GTGACTGGAGTTCAGACGTGTGCTCTTCCGATCTCTCGGCTGCGTTCTTCATCGATGC |
| AgITS2R4 | GTGACTGGAGTTCAGACGTGTGCTCTTCCGATCTACTCGGCTGCGTTCTTCATCGATGC |
| AgITS2R5 | GTGACTGGAGTTCAGACGTGTGCTCTTCCGATCTGACTCGGCTGCGTTCTTCATCGATGC |
| AgITS2R6 | GTGACTGGAGTTCAGACGTGTGCTCTTCCGATCTTGACTCGGCTGCGTTCTTCATCGATGC |
| **PCR2** | |
| Forward | AATGATACGGCGACCACCGAGATCTACACTCTTTCCCTACACGACGCTCTTC |
| Reverse (barcoded) | GTGACTGGAGTTCAGACGTGTGCTC TTCCGATCT NNNN GA TCCTCCGCTTATTGATATGC |

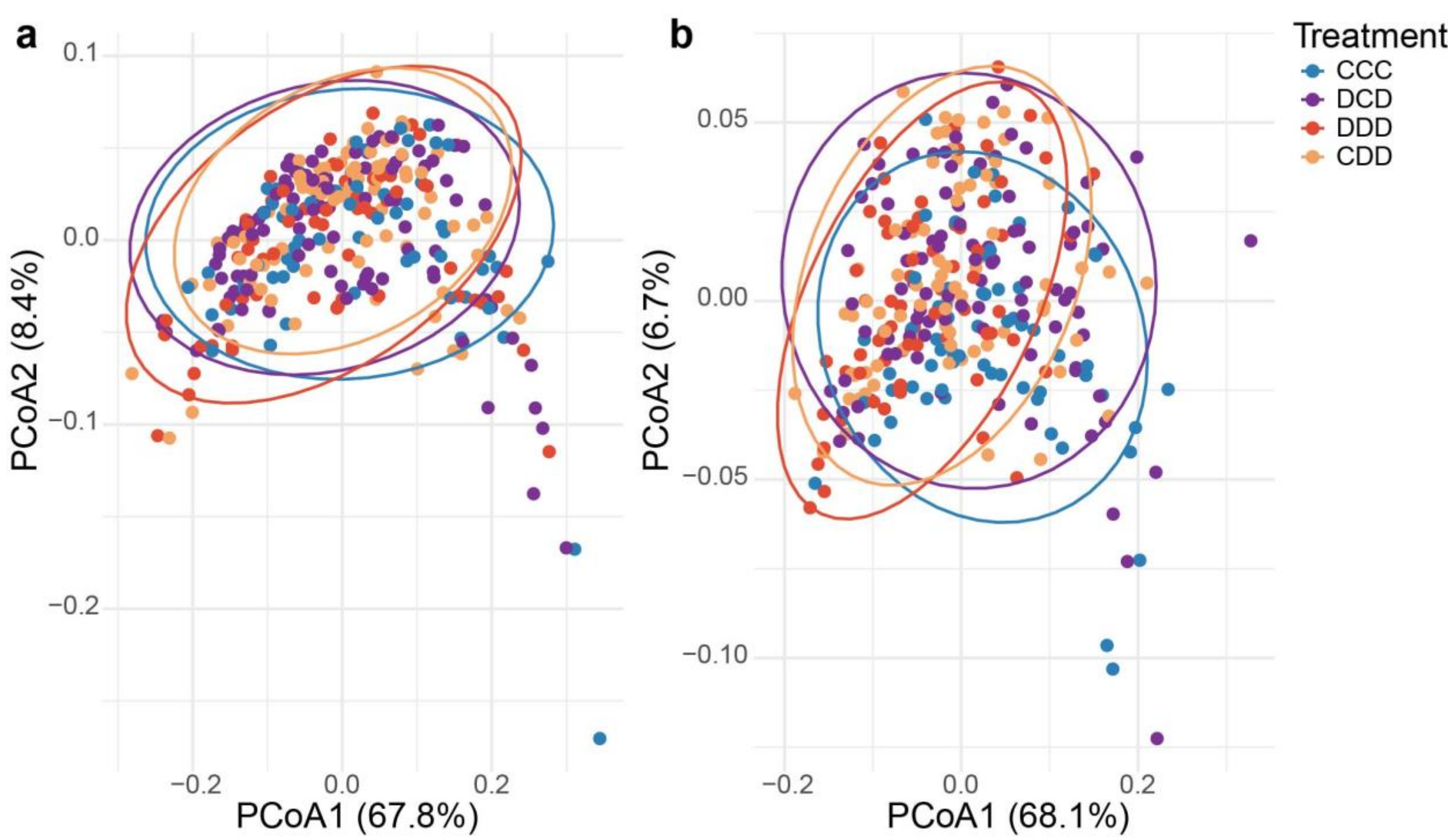


**Fig. S1. Multivariate patterns of *Populus tremuloides* foliar chemistry across drought treatments.** Principal coordinates analysis (PCoA) of foliar chemical profiles based on UPLC

peak intensities normalized by sample starting weight for (A) 2022 and (B) 2023. Points represent individual samples colored by treatment, and ellipses indicate group-level dispersion.

The first two PCoA axes explained similar proportions of variance in each year (2022: 67.8% and 8.4%; 2023: 68.1% and 6.7%). Samples showed partial separation by treatment, with substantial overlap among groups.

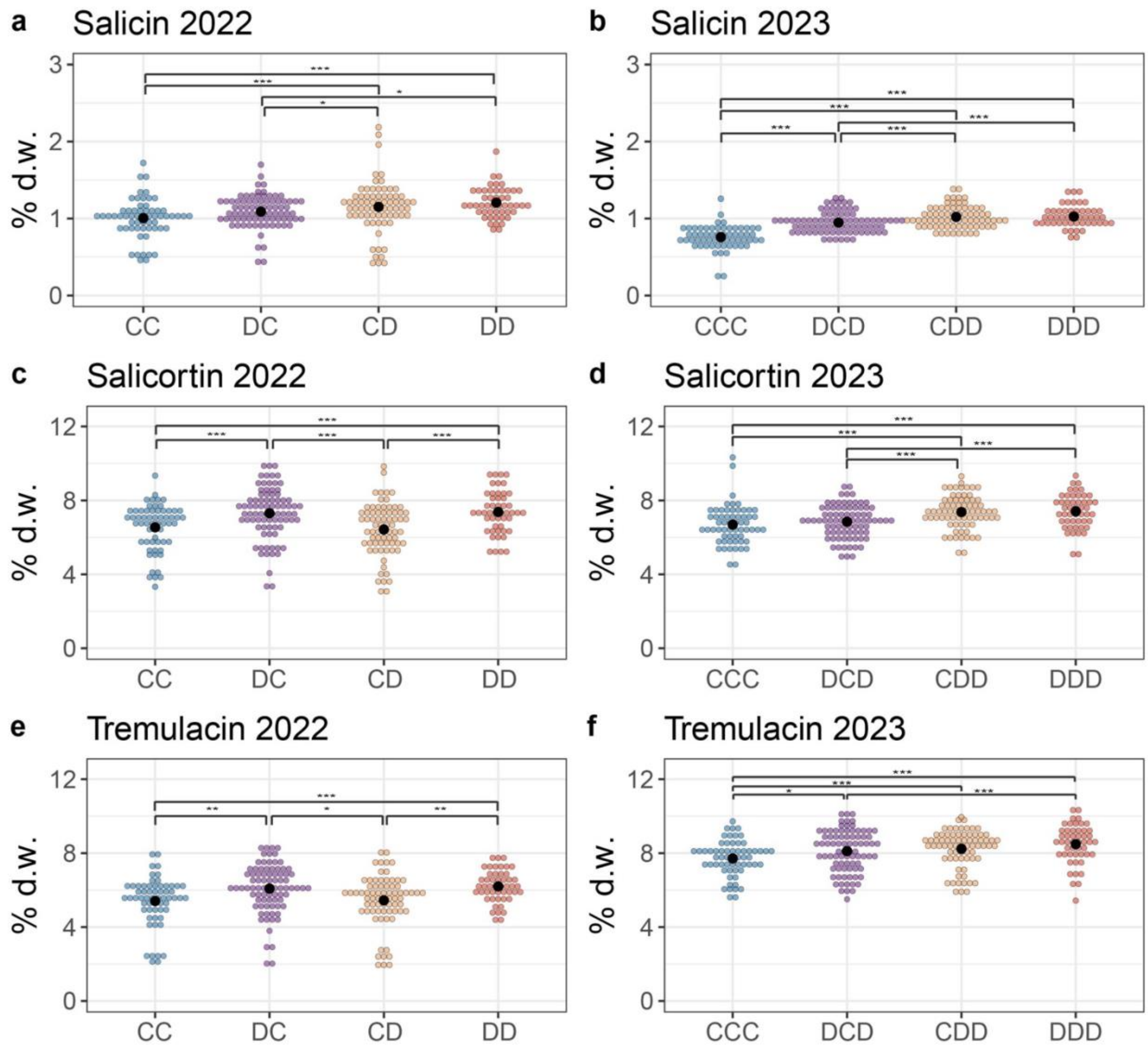


**Fig. S2. Drought legacy effects on *P. tremuloides* allocation to individual salicinoid phenolic glycosides (SPGs).** Percent dry weight (% d.w.) allocation to salicin (**a,b**), salicortin (**c,d**), and tremulacin (**e,f**) in 2022 (**a,c,e**) and 2023 (**b,d,f**). Colored points represent individual samples; black symbols indicate means ± SE.

Pairwise comparisons were performed using estimated marginal means (emmeans) with Tukey-adjusted p-values (*** = p< 0.001, ** = p < 0.01, * = p < 0.05). Sample sizes were n = 51 (CCC, DDD) and n = 74 (CDD, DCD).

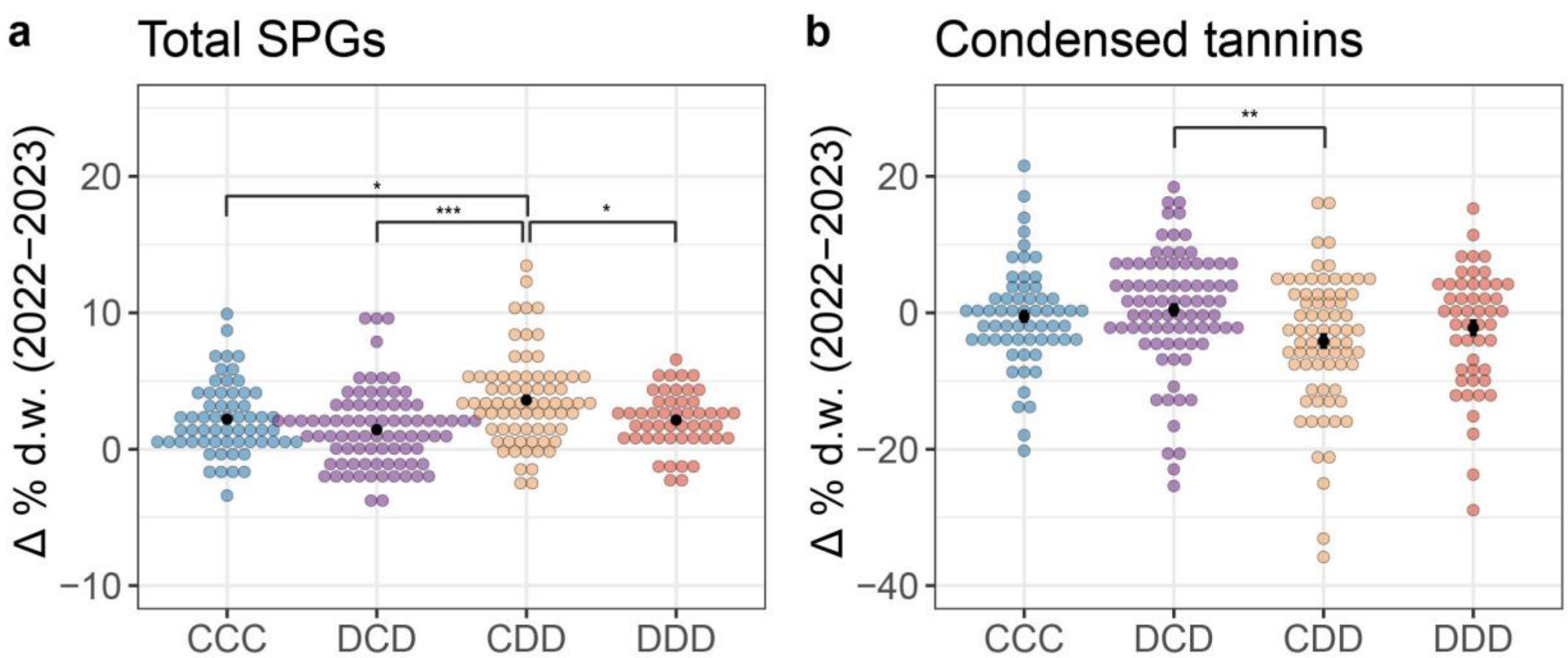


**Fig. S3. Changes in allocation to *P. tremuloides* defensive chemistry differ among drought treatments.** Change in percent dry weight (Δ % d.w.) from 2022 to 2023 for total salicinoid phenolic glycosides (SPGs; **a**) and condensed tannins (CTs; **b**). Colored points represent individual samples; black points and bars indicate means ± SE.

Pairwise comparisons were performed using estimated marginal means (emmeans) with Tukey-adjusted p-values (*** = p< 0.001, ** = p < 0.01, * = p < 0.05). Sample sizes were n = 51 (CCC, DDD) and n = 74 (CDD, DCD).

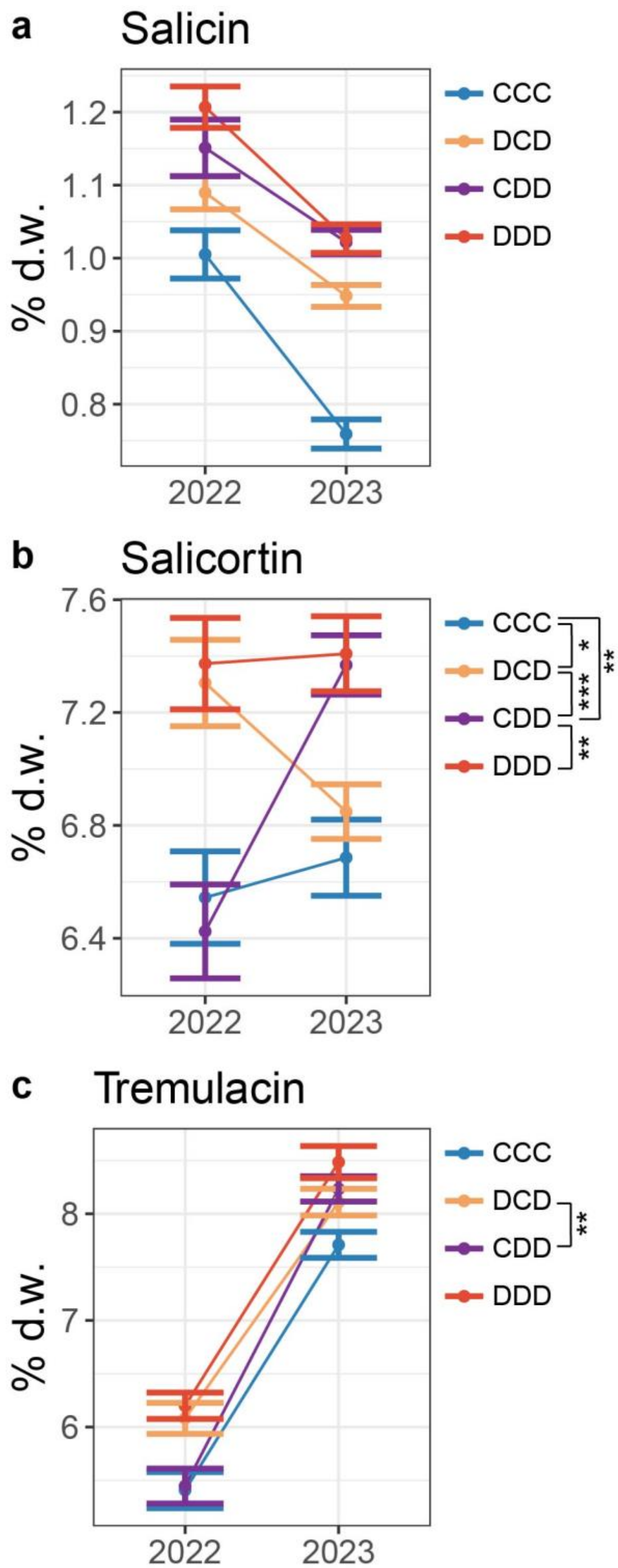


**Fig. S4. Drought legacy effects on individual salicinoid phenolic glycosides (SPGs) in *P. tremuloides*.**
Mean percent dry weight (% d.w.) allocation to salicin (**a**), salicortin (**b**), and tremulacin (**c**) in 2022 and 2023 across drought treatments. Points represent means, and error bars indicate ± SE.

Post hoc comparisons of change from 2022 to 2023 were performed using estimated marginal means (emmeans) with Tukey-adjusted p-values (*** = p< 0.001, ** = p < 0.01, * = p < 0.05), consistent with analyses presented in Fig. 3 and S4.

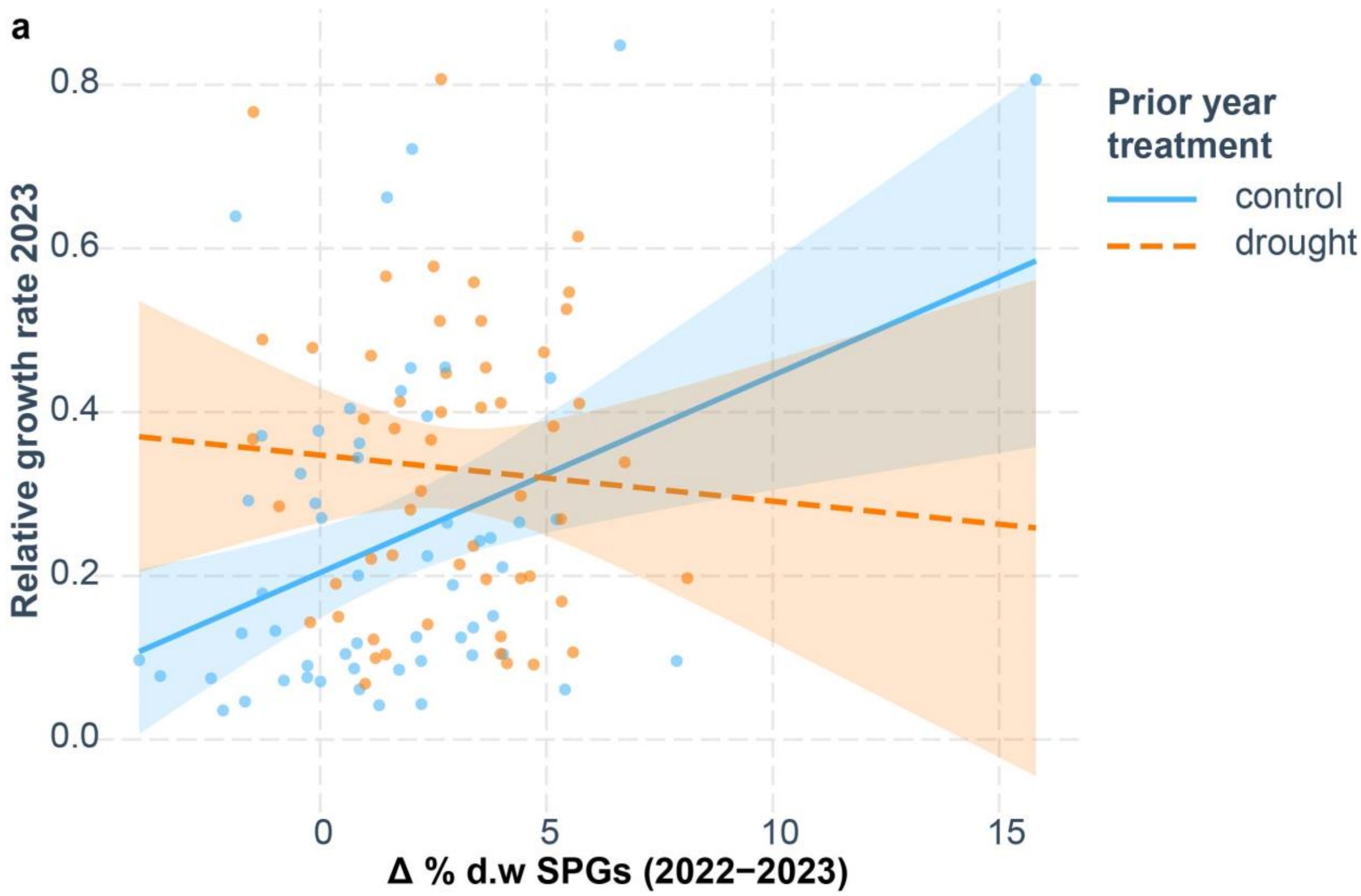


**Fig. S5. Relationship between *P. tremuloides* salicinoid phenolic glycoside (SPG) allocation and relative growth rate depends on prior-year drought treatment**. Associations between changes in SPG allocation and relative growth rate (RGR) in 2023 differed by prior-year treatment. Increased SPG allocation was associated with higher RGR in trees that were well-watered in the prior year (β = 0.0241, 95% CI: 0.0084–0.0398), but not in trees that experienced prior-year drought (β = −0.0056, 95% CI: −0.0287–0.0175).

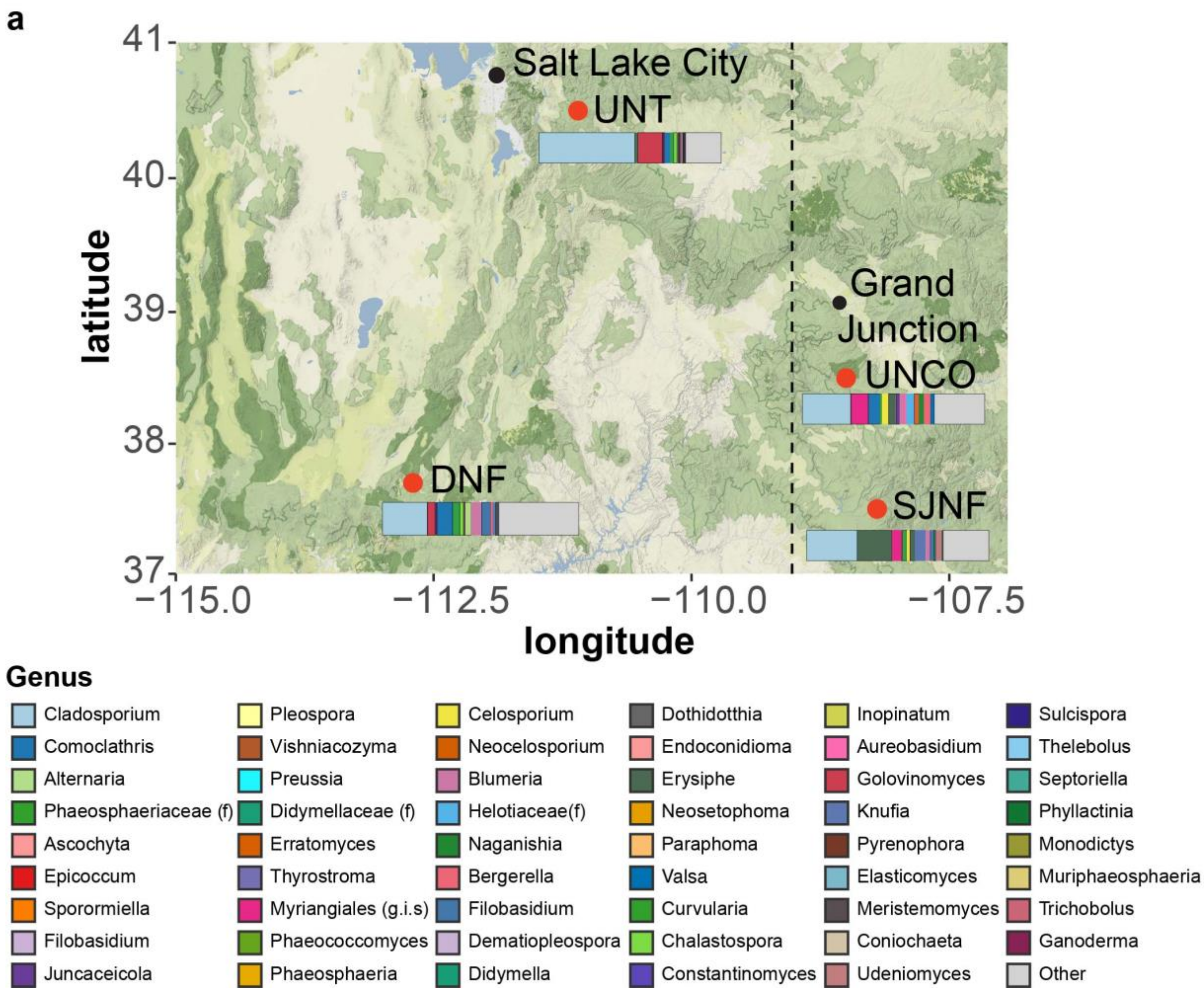


**Fig. S6. Relative abundance of dominant fungal genera across field sites in 2023.** Map of field sampling locations in the western United States with stacked bar plots showing mean relative abundance of dominant fungal genera in *P. tremuloides* leaves at each site. Bars represent genus-level composition averaged across samples within each site; colors correspond to genera as indicated in the legend.

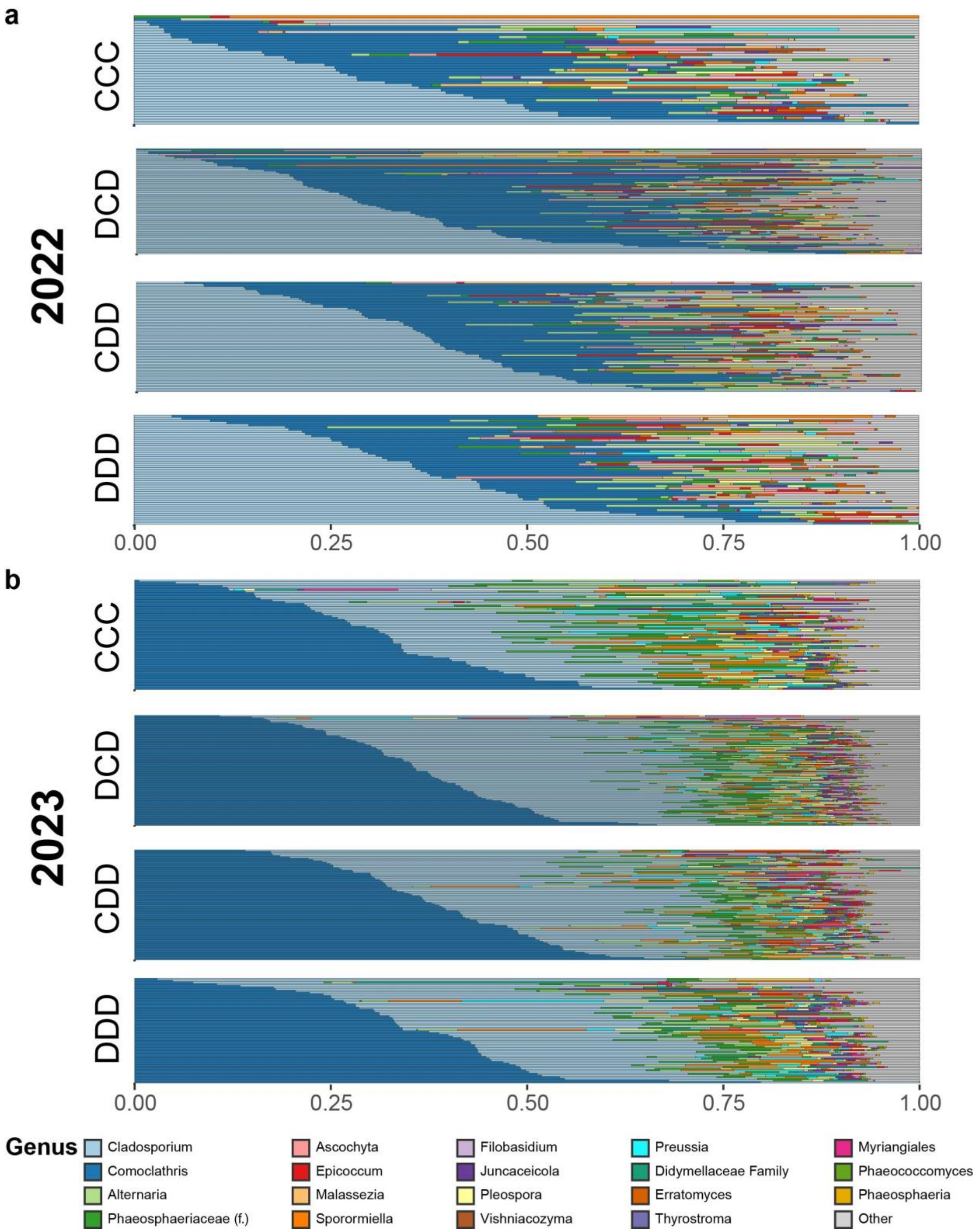


**Fig. S7. Genus-level composition of foliar fungal communities across drought treatments in the common garden.** Stacked bar plots showing relative abundance of fungal genera for

individual *P. tremuloides* leaf samples in 2022 (**a**) and 2023 (**b**), grouped by treatment. Each bar represents a single sample, and colors indicate genus-level taxonomic assignments.

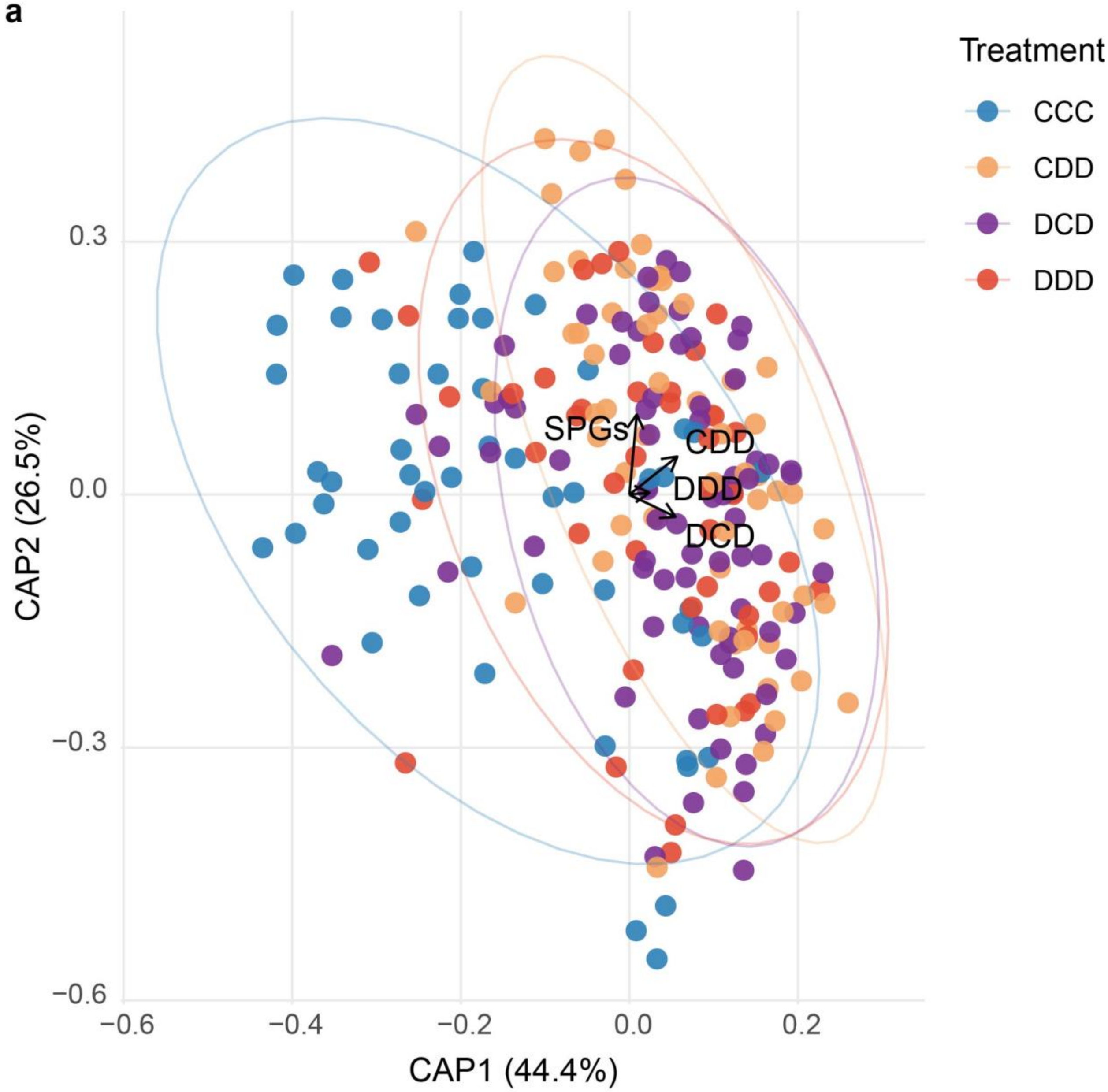


**Fig. S8. Constrained ordination of foliar fungal communities from *P. tremuloides* in the common garden (2023).** Distance-based redundancy analysis (dbRDA) of fungal community composition showing separation among drought treatments. Points represent individual samples, and ellipses indicate group-level dispersion. Vectors represent fitted environmental variables, including total SPGs, with direction indicating the gradient of increasing values.

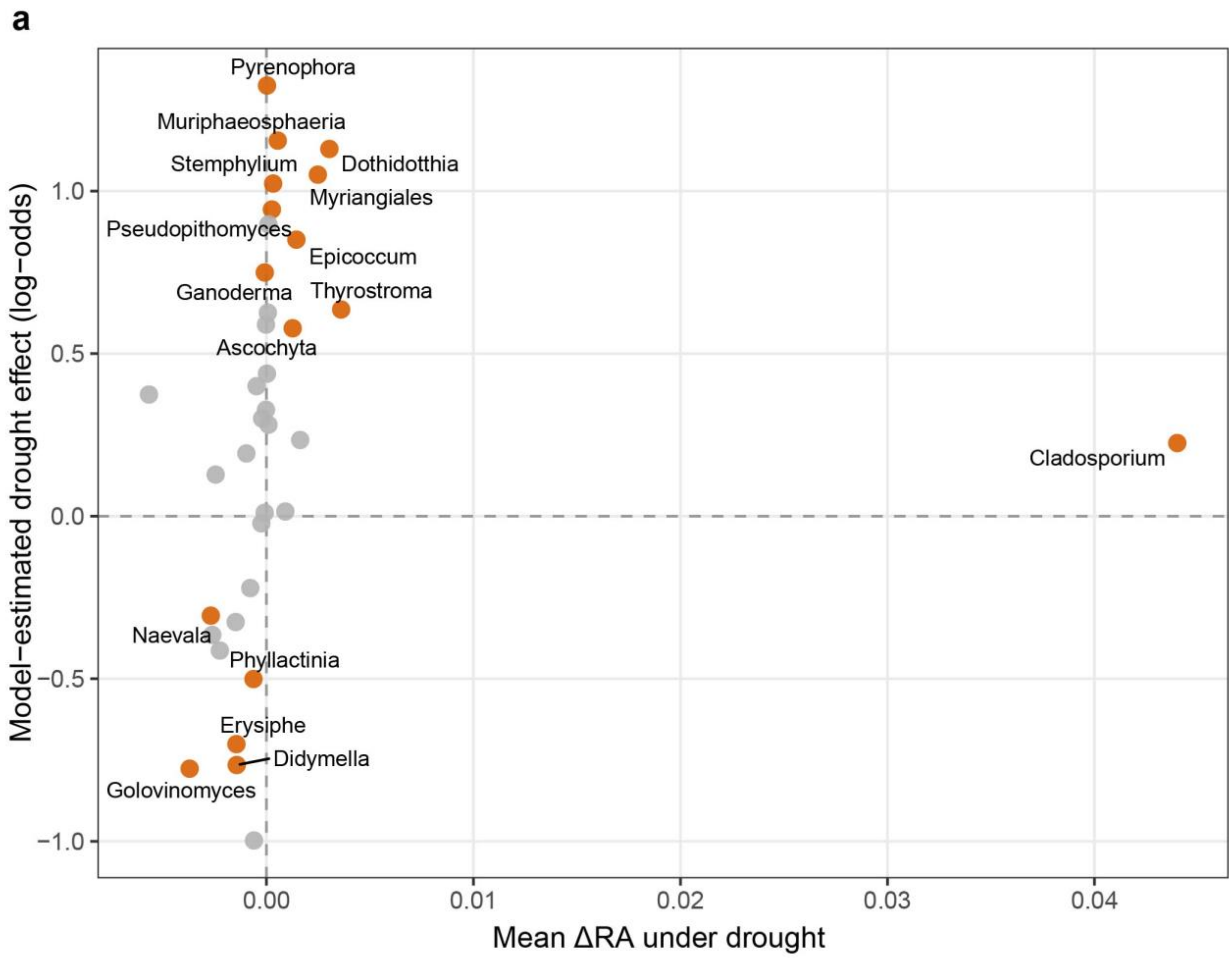


**Figure S9. Concordance between model-estimated drought effects and observed changes in relative abundance of fungal genera in common garden aspen (*P. tremuloides*).** Points represent fungal genera, with the x-axis showing the mean change in relative abundance under drought (ΔRA) and the y-axis showing model-estimated drought effects (log-odds) from genus-level beta-binomial GLMMs. Points highlighted in orange indicate genera identified as significantly responsive to drought in model-based analyses. Positive values indicate increases under drought.

**Table S2. Beta-binomial GLMM results for taxa most associated with drought-stressed *P. tremuloides* in the common garden with corresponding results from the comparison between the driest (Uncompahgre National Forest (UNCO)) and wettest (Uinta National Forest (UNT)) sampled field sites.**

| Genus | garden drought effect | adj. p-value | SE | UNCO - UNT | p-value | SE |
|---|---|---|---|---|---|---|
| *Dothidotthia* | 1.1294 | 0.0002 | 0.3010 | -0.8459 | 0.3922 | 0.4783 |
| *Myriangiales* | 1.0502 | > 0.0001 | 0.2166 | 0.7720 | 0.0427 | 0.2773 |

| | | | | | | |
|---|---|---|---|---|---|---|
| *Stemphylium* | 1.023 | 0.0001 | 0.2639 | -0.4122 | NA | NA |
| *Epicoccum* | 0.8505 | 0.0023 | 0.2794 | -0.4445 | 0.9862 | 0.8673 |
| *Ganoderma* | 0.7495 | 0.0606 | 0.3993 | 25.1492 | 1.0000 | 132977.8532 |
| *Thyrostroma* | 0.6356 | 0.0103 | 0.2476 | 1.2623 | 0.5580 | 0.8377 |
| *Sulcispora* | 0.6258 | 0.189 | 0.4764 | -1.5612 | 1.0000 | 41289.7381 |
| *Curvularia* | 0.3998 | 0.1065 | 0.2477 | 0.3080 | 0.9981 | 1.0005 |
| *Cladosporium* | 0.2248 | 0.0145 | 0.0920 | -1.0071 | > 0.0001 | 0.1820 |
| *Erysiphe* | -0.7011 | 0.0012 | 0.2164 | -0.5025 | 0.9560 | 0.7155 |
| *Didymella* | -0.7654 | 0.0588 | 0.4050 | 1.0224 | 0.9199 | 1.2251 |
| *Golovinomyces* | -0.7767 | >0.0001 | 0.1952 | -3.6579 | > 0.0001 | 0.4406 |

NA = Not available

**Supplementary References**

**Kerr KL, Fickle JC, Anderegg WRL**. **2023**. Decoupling of functional traits from intraspecific patterns of growth and drought stress resistance. *The new phytologist* **239**: 174–188.
